# Poly(ethylene-*co*-vinylalcohol)/Poly(δ-valerolactone)/Aspirin Composite: Model for a New Drug-Carrier System

**DOI:** 10.3390/polym11030439

**Published:** 2019-03-06

**Authors:** Abdulaziz Ali Alghamdi, Waseem Sharaf Saeed, Abdel-Basit Al-Odayni, Fahad A. Alharthi, Abdelhabib Semlali, Taieb Aouak

**Affiliations:** 1 Chemistry Department, College of Science, King Saud University, P.O. Box 2455, Riyadh 11451, Saudi Arabia; aalghamdia@ksu.edu.sa (A.A.A.); aalodayni@ksu.edu.sa (A.-B.A.-O.); fharthi@ksu.edu.sa (F.A.A.); 2 Groupe de Recherche en Écologie Buccale, Faculté de Médecin Dentaire, Université Laval, G1V 0A6, QC, Canada; abdelhabib.semlali@greb.ulaval.ca

**Keywords:** poly(ethylene-*co*-vinylalcohol)/poly(δ-valerolactone)/aspirin, composite, control of drug release, the dynamic mechanical properties, effect of the degree of swelling, diffusion mechanism

## Abstract

The release dynamics of aspirin(ASP), used as a drug model, from the poly(ethylene-*co*-vinyl alcohol)/poly(δ-valerolactone) (PE-*co*-VAL/Pδ-VL) hydrogel blend was controlled by varying the blend’s degree of swelling through a gradual loading of Pδ-VL (hydrophobic polymer) in this copolymer matrix. To achieve this goal, a series of PE-*co*-VAL/Pδ-VL blends with different ratios was prepared through the solvent casting method, and the miscibility of this polymer blend was evaluated by using Fourier transform infrared spectroscopy, differential scanning calorimetry, X-ray diffraction, and scanning electronic microscopy methods. The tests of cell adhesion and growth on the PE-*co*-VAL/Pδ-VL specimens were performed using the 3-(4,5-demethylthiazol-2-yl)-2,5-diphenyltetrazolium bromide (MTT) method and the results obtained were the best performance in terms of cell viability, cell adhesion, and growth of the PE-*co*-VAL/Pδ-VL50 material. The dynamic mechanical properties of the prepared material were also examined by dynamic mechanical analysis; the results obtained showed a material having intermediary mechanical properties between those of the two components. On the basis of these characterizations, the blend showing the best performance, such as the PE-*co*-VAL/Pδ-VL50 system, was chosen as a carrier to study the in vitro control of the release dynamics of ASP from the ASP/PE-*co*-VAL/Pδ-VL drug-carrier system when administered orally, in which the influences of the ASP content and the degree of swelling of the PE-*co*-VAL/Pδ-VL blend were investigated. Based on the data obtained and the gastrointestinal transit time reported by Beltzer et al., it was possible to estimate the distribution of the in vitro cumulative ASP released in different digestive system organs regardless of the actions of any enzymes and microorganisms and select the best-performing drug-carrier system.

## 1. Introduction

In the past decade the development of new biocompatible and biodegradable polymers has received considerable attention from many researchers working in the biomedical field worldwide [1,2,3,4,5,6,7,8]. Many works have been reported in the literature on the enhancement of certain key properties that adapt with the conditions of the specific environment in which such polymers operate. Recently, pH-sensitive hydrogels, such as poly(acrylic acid) (PAA) [9], poly(methacrylic acid) [10], poly(vinylpyrolidone/acrylic acid [11], poly(vinylalcohol) [12], poly(acrylamide-*co*-methylmethacrylate) [13], poly(acrylamide-2-acrylamido-2-methyl-1-propanesulfonic acid) [14], and other methacrylic-type polymeric prodrugs, have become more attractive in the drug delivery domain. The substances produced from these polymers, when crosslinked, swell in water or aqueous solutions below the lower critical solution temperature (LCST) and shrink above the LCST. These substances, when fully swollen, have unique properties, such as softness, elasticity, and low interfacial tension with water and biological fluids. The properties of polymeric hydrogels, especially of natural hydrogels, make them similar to the extracellular matrix in living tissues. For hydrogels, there is less risk of a negative immune response because of their low interfacial tension with body fluids, which reduces cell adhesion. Many hydrogels show improved tissue permeabilities and drug residence times because of their muco-adhesive and bio-adhesive properties, which make them very good vehicles for drug delivery [15,16]. Generally, hydrogels based on natural polymers are nontoxic, biodegradable, biocompatible, abundant, and cheap to manufacture. However, their poor mechanical properties considerably limit their applications in the biomedical domain. Biocompatible synthetic hydrogels can have excellent mechanical strengths but are expensive and non-biodegradable.

Poly(ethylene-*co*-vinyl alcohol) (PE-*co*-VAL) is a synthetic, random, and semi-crystalline copolymer over the entire range of its composition in spite of the irregularity and non-stereo-specificity of vinyl alcohol units distributed in the copolymer chain [17]. This nontoxic copolymer is endowed with excellent mechanical properties and is widely used in food packaging. However, it contains low ethylene units and is susceptible to complete biodegradation in the presence of enzymes [18,19,20]. The presence of ethylene units in PE-*co*-VAL lowers its ability to swell, thereby making this substance a good carrier for drug delivery. This feature is doubtless a key element in the field of drug delivery; however, it is still not enough, because it does not permit to control the release parameters because of the uncontrollability of the ratio of the ethylene/vinyl alcohol units and their random distribution.

Acetylsalicylic acid, also called aspirin (ASP), is widely used as a nonsteroidal anti-inflammatory drug. It is known for prolonging bleeding time and effectively inhibiting platelet aggregation. When is administrated orally, this drug also has an undesirable effect of direct irritation of the gastric mucosa caused by the inhibition of prostaglandins and prostacyclins, thus causing ulceration, epigastric distress, and/or hemorrhage [21]. The extended release of an ASP formulation improves patient compliance, reduces many of the undesirable side effects, and reduces the frequency of its administration [22]. Thus, when a selected polymer-drug system is orally administered, it must meet the following conditions: the drug carrier system should not be toxic; drugs must be well-protected in unstable biological environments, including gastrointestinal tract-induced degradation and early liver effects after oral administration before reaching targeted sites [23]; have a possibility of evenly releasing the largest amount of medication directly into the target location or that have the function of absorbing digestive juice (intestines); show good drug-carrier adhesion to organ cells to prolong drug release time in the targeting area; and have adaptable mechanical properties.

Only a few works have been reported in the literature in this field, such as that investigated by Young et al. [24], on the PE-*co*-VAL copolymer used as a carrier and doxorubicin as a model drug. The results obtained revealed that the release of doxorubicin from this drug-carrier system happens in two steps. The first step is characterized by a rapid dynamic attributed to the presence of ASP aggregated in the macroscopic pores on the surface of the material, which diffuses rapidly. The second step is characterized by a slow and prolonged release. In a study reported by Alotaibi et al. [25,26] on the release of ASP from ASP grafted to, or mixed with, PE-*co*-VAL, it was found that although this polymer gave very encouraging results in terms of the release stability in neutral pH media, a considerable amount of ASP was released in a very short time (40–60 wt% in 6 h) owing to its high degree of swelling. A reduction in the capability of a polymer hydrogel to swell in a certain pH media can be easily obtained by blending the polymer with another hydrophobic polymer, provided that the two are miscible. Indeed, this technique can be an alternative, in which control on the swelling property can be easily exercised by controlling the hydrophilic/hydrophobic ratio. This method, which has been proved very effective in solving many of the problems encountered in the field of organic materials [27], is easily realizable, undoubtedly very economical, and precise.

In this work, the control of the release dynamics of ASP, used as a drug model, from the PE-*co*-VAL hydrogel, was carried out by varying its degree of swelling through a gradual incorporation of poly(δ-valerolactone) (Pδ-VL), a hydrophobic polymer, into this copolymer. To achieve this goal, a series of PE-*co*-VAL/Pδ-VL blends with different ratios was prepared by using the solvent casting method, and the miscibility of these pairs of polymer were studied through Fourier transform infrared (FTIR), differential scanning calorimetry (DSC), X-ray diffraction (XRD), and scanning electronic microscopy (SEM) methods. The tests of the cell adhesion and growth on the PE-*co*-VAL/Pδ-VL specimens were undertaken through the 3-(4,5-demethylthiazol-2-yl)-2,5-diphenyltetrazolium bromide (MTT) method. The effect of the Pδ-VL content on the degree of swelling of the PE-*co*-VAL/Pδ-VL blend was then investigated. The dynamic mechanical properties of the prepared material were also examined through dynamic mechanical analysis (DMA). On the basis of these characterizations, the blend showing the best performance was then used as a carrier to study the in vitro control of the release dynamics of ASP from the ASP/PE-*co*-VAL/Pδ-VL drug-carrier system when administered orally, in which the influences of the ASP content and the degree of swelling of the PE-*co*-VAL/Pδ-VL blend were investigated, and the estimated distribution of the cumulative ASP released from the ASP/PE-*co*-VAL/Pδ-VL50 drug-carrier system on the principal digestive organs was tabulated.

## 2. Materials and Methods

### 2.1. Chemicals

PE-*co*-VAL containing 32 mol% of ethylene units with a molar mass of 20,000 g·mol^−1^ and δ-VL (purity 98%) were procured from Sigma-Aldrich (Taufkirchen, Germany). LoVo cells, Dulbecco’s modified Eagle’s medium, and fetal bovine serum were purchased from the ATCC company (Manassas, VA, USA). DMF (purity 97%) was procured from (Panreac Química SLU, Castellar del Vallès, Spain). All these substances were used without further purification, except for δ-VL, which was dehydrated under dehydrated calcium chloride and purified by distillation under vacuum.

### 2.2. Polymerization of δ-Valerolactone

Pδ-VL was obtained through ring opening polymerization using concentrated HCl as a catalyst according to the method described in the literature [28]. The molar mass of the resultant Pδ-VL was evaluated in THF at 30 °C by size exclusion chromatography (SEC) at ambient temperature (~25 °C) on a Varian instrument equipped with a JASCO type 880-PU HPLC pump, refractometer, UV detectors, and TSK gel columns calibrated with polystyrene standards. The results obtained indicated an average molar mass of 1.7 × 10^4^ g·mol^−1^ and a dispersity of 2.6.

### 2.3. Preparation of PE-co-VAL/Pδ-VL Blends

In a 100 mL flask, a known amount of PE-*co*-VAL was completely dissolved under continuous stirring in 10 mL of DMF at 80 °C to prepare polymeric solution-A. In another 100 mL vial, a known amount of Pδ-VL was completely dissolved at 30 °C, under the same conditions as those of solution A, forming solution B. The two resulting binary systems were mixed together to form the PE-*co*-VAL/Pδ-VAL/DMF ternary solution, which was then poured onto a Teflon plate and allowed to air dry for one day and then kept under vacuum at 40 °C until the mass of the blend was constant. Various PE-*co*-VAL/Pδ-VL systems containing 10, 25, 50, 75, and 90 wt% of Pδ-VL were prepared through the same method. The preparation conditions are listed in Table 1.

### 2.4. Preparation of ASP/PE-co-VA/Pδ-VL50 Composites

A know amount of ASP was added to the PE-*co*-VAL/Pδ-VL50/DMF ternary system containing equal amount of components, prepared as described in the Section 2.3, at 50 °C under continuous stirring until complete dissolution, thereby forming a limpid and very viscous solution. The ASP/PE-*co*-VAL/Pδ-VL50/DMF quaternary solution was then poured onto a horizontal Teflon plate and left to dry at ambient temperature for 48 h followed by drying in a vacuum oven at 40 °C. The dryness was monitored through the stability of the material weight. A series of ASP/PE-*co*-VAL/Pδ-VL50 blends containing 2, 5, 7, and 10 ASP contents were prepared through this method. The preparation conditions are listed in Table 2.

### 2.5. Aspirin Release Experiment Setup

Approximately 50 mg of the ASP/PE-*co*-VA/Pδ-VL50film was immersed in 40 mL of the release medium (pH values of 1, 2, 3, or 7) in a closed 100 mL flask. The flask was then maintained under moderate stirring at 37 °C by means of a thermostatic bath. To analyze the ASP amount released, aliquots of 2 mL of the medium were taken at different time intervals and kept in a closed vessel for concentration measurement afterward. To avoid any equilibrium between the concentrations of ASP inside and outside the polymer matrix during the release process, and thereby gradually reduce the dynamic release of ASP from the specimen, the withdrawn 2 mL was immediately replaced by the same volume of the fresh medium after each time interval. Importantly, note that the pH of the medium was not affected by the small amount released of ASP, meaning that the addition of traditional buffer to the medium was, in our case, not necessary.

### 2.6. Aspirin Concentration Measurement

The concentrations of ASP released in different pH media from the ASP/PE-*co*-VAL/PδVL50specimen were measured through spectrophotometry by means of a Hitachi U-2910 spectrophotometer at a wavelength of 275 nm corresponding to the maximal absorbance of the ASP phenyl group using the ASP calibration curve obtained in the respective medium.

### 2.7. Swelling Behavior

The study of the swelling behavior of a hydrogel is an essential element to understand the dynamics of a drug released from a drug/polymer system. According to various studies, this property is affected by different parameters such as the cross-link degree of polymer [29,30], temperature [31,32], and pH of the medium in which the drug will be released [33,34]. In this work, PE-*co*-VAL/Pδ-VL film specimens were dried under vacuum at 60 °C for two days. Four pieces of each film having practically the same dimensions were immerged in media of pH values of 1, 3, 5, and 7 maintained at body temperature (37°C) until swelling equilibrium (saturation) was reached. The degree of swelling of a polymeric material (*S*) in a pH medium is calculated with the help of Equation (1):

(1) $$S(\%)=\frac{w_{t}-w_{o}}{w_{o}}\times 100$$

where *w_o_* and *w_t_* are the weights of the specimen before and after *t* time of the swelling process, respectively.

### 2.8. Characterization

The FTIR analyses of ASP/PE-*co*-VAL/Pδ-VL50 system and its components were carried out at 25 °C using a Perkin Elmer 1000 spectrophotometer. All scans were performed on transparent and sufficiently thin films, and the spectra were obtained with an accuracy of 2 cm^−1^. The glass transition temperatures and the melting temperatures of polymeric materials and pure ASP were measured on a Shimadzu DSC 60A system, which was previously calibrated with indium. Between 8 and 10 mg of the polymer or blend was deposited in an aluminum pan and then closed before being placed in the DSC analysis cell. All samples were scanned from −100 to +200 °C under an atmosphere of nitrogen gas at a heating rate of 20 °C·min^−1^. All the thermograms taken from the second scan run revealed no traces of degradation. The *T_g_* value of pure constituents or their mixture was taken precisely as the median point on the part of the thermogram indicating the variation in the heat capacity with the temperature. The *T_m_* value was taken exactly at the top of the endothermic peak.

The crystalline structures of pure ASP, PE-*co*-VAL, and Pδ-VL were examined through XRD analysis on an X-ray diffractometer (RigakuDumax 2000) equipped with a Cu anode tube, with a tube voltage of 40 KV/40 mA and generator current of 100 mA. All samples were examined at 2θ = 5°–50° at a scanning rate of 1.0°·min^−1^. The surface morphologies of the polymer and nanocomposites were examined on a JEOL JSM 6360 scanning electronic microscope with acceleration voltages of 3 and 5 kV. To reduce any buildup deposited on the film surfaces, specimens were carefully coated with a thin layer of gold with the help of a JEOL JFC-1600 Auto Fine Coater operated at 20 mA for 80 s prior to the analysis. The cell adhesion and cell proliferation tests of the PE-*co*-VAL, Pδ-VL, and PE-*co*-VAL/Pδ-VL specimens were performed using an MTT assay, as conducted previously by Semlali et al. [35,36]. Briefly, the tests were performed on 12-well plates, of which one well was treated by collagen (5 mg·mL^−1^) as a positive control of adhesion. Using a sterile technique, each material disk (15-mm diameter) was placed at the bottom of a well and exposed to 10×10^3^ LoVo cells in 1.0 mL of 10% FBS-supplemented DMEM medium, and immediately incubated in a 5%-CO_2_ humid atmosphere at 37 °C for 24 h for the adhesion test and 24h and 48 h for the cell growth test. After incubation, the material disks populated with LoVo cells were cultured in the presence of 1.0 % (*v*/*v*) MTT solution (5 mg·mL^−1^) for 24 h and 48h before stopping the reaction. At the end of incubation, the test materials were washed twice with sterile PBS to remove the non-adhering cells, and then 500 µL of 0.04-N isopropanol solution was added to each well. The dissolved material was transferred from each well to a new 96-well flat-bottom plate and measured spectrophotometrically at 550 nm using an ELISA reader (Model 680, BioRad Laboratories, Mississauga, ON, Canada). The percentage of adhesion was calculated by Equation (2):

(2) $$Cell\;Adhesion\;\left(\%\right)=\frac{DO_{s}-DO_{nc}}{DO_{pc}-DO_{nc}}\;\times \;100$$

where DO_s_, DO_pc_, and DO_nc_ are the optical densities of the test sample, positive control, and negative control, respectively.

## 3. Results and Discussions

### 3.1. Assessment of Cell Adhesion and Growth

**Figure 1:**
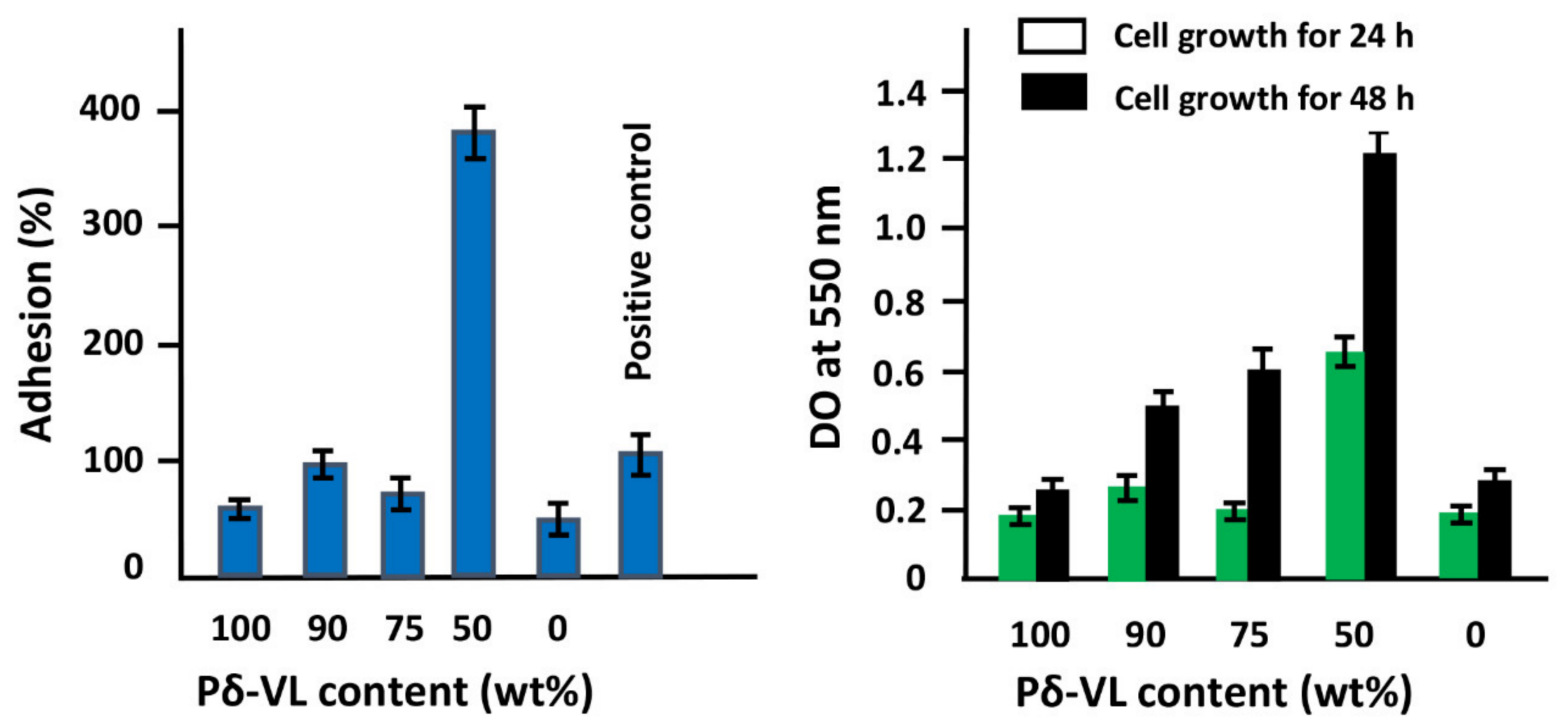
Cell adhesion and growth on the pure Pδ-VL and PE-*co*-VAL/Pδ-VL blend specimens with different Pδ-VL contents. The error bars correspond to the standard deviation between the minimal and the maximal values obtained from four experiments. The positive control is the collagen membrane (collape) (adhesion 100%). The error bars correspond to the standard deviation between the minimum and maximum values obtained from the four experiments carried out.

Figure 1 presents the results of the cell adhesion and growth screening tests performed with PE-*co*-VAL/Pδ-VL blends with different Pδ-VL contents. This figure reveals that the LoVo cell adherence after 24 h of culture was more visible on the blend containing an equal ratio of PE-*co*-VAL and Pδ-VL contents (PE-*co*-VAL/Pδ-VL50) compared with those of the pure components (Figure 1a). Moreover, the specific growth during both the 24 and 48 h culture periods reflected a higher growth rate on this same material compared with the other tested specimens (Figure 1b).The increase in adhesion and growth on the PE-*co*-VAL/Pδ-VL50 specimen surface is probably caused by an increase in the density of the porosity on the surface of this polymeric system and an increase in its wettability caused by an increase in the hydrogen bonds created between (i) the water molecules and the vinyl alcohol units of PE-*co*-VAL and (ii) the vinyl alcohol units and the cells. According to the investigation reported by Abdelwafa et al. [37], the formation of functional groups such as hydroxyl or carboxyl groups on a scaffold promotes cell proliferation in a protein-mediated cell adhesion mechanism. The best performance in terms of cell viability, cell adhesion, and growth makes the PE-*co*-VAL/Pδ-VL50 material an important potential candidate for use as a vector in the field of drug delivery.

### 3.2. FTIR Analysis

**Figure 2:**
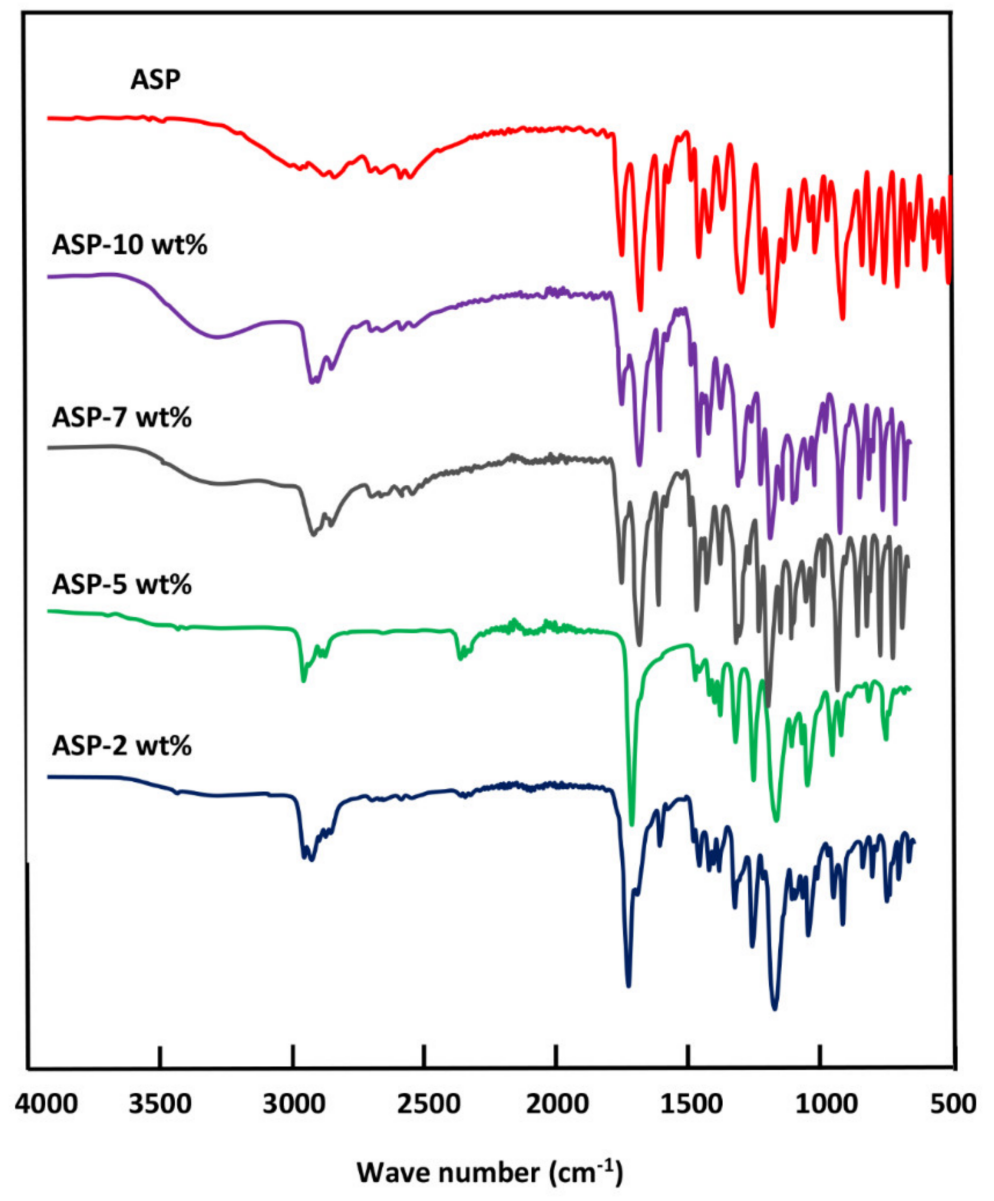
FTIR spectra of ASP and ASP/PE-*co*-VAL/Pδ-VL50 drug carrier systems containing different ASP loads.

**Scheme 1:**
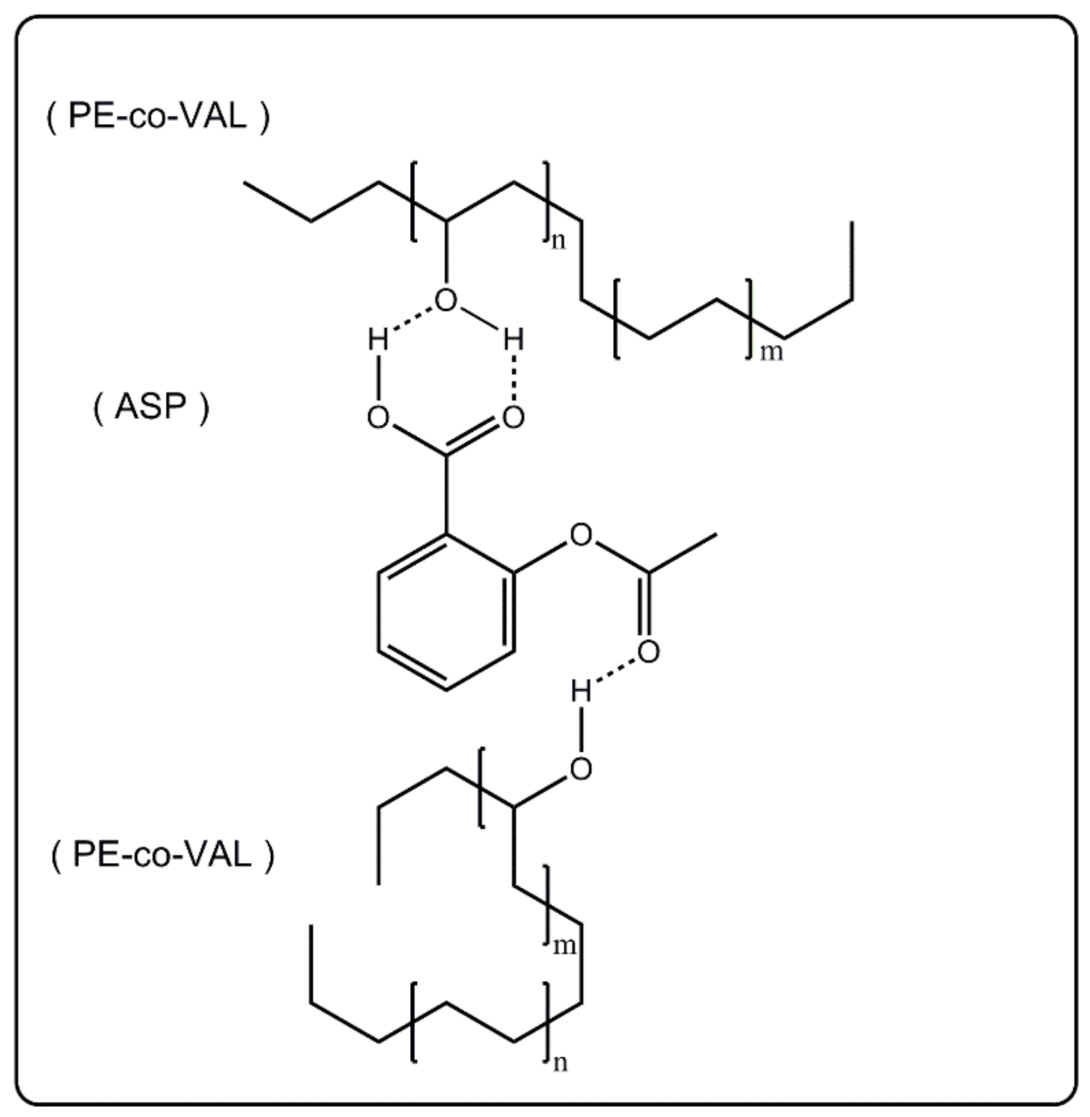
Hydrogen bond interactions between the hydroxyl groups of PE-*co*-VAL and the carbonyl groups of ASP.

**Scheme 2:**
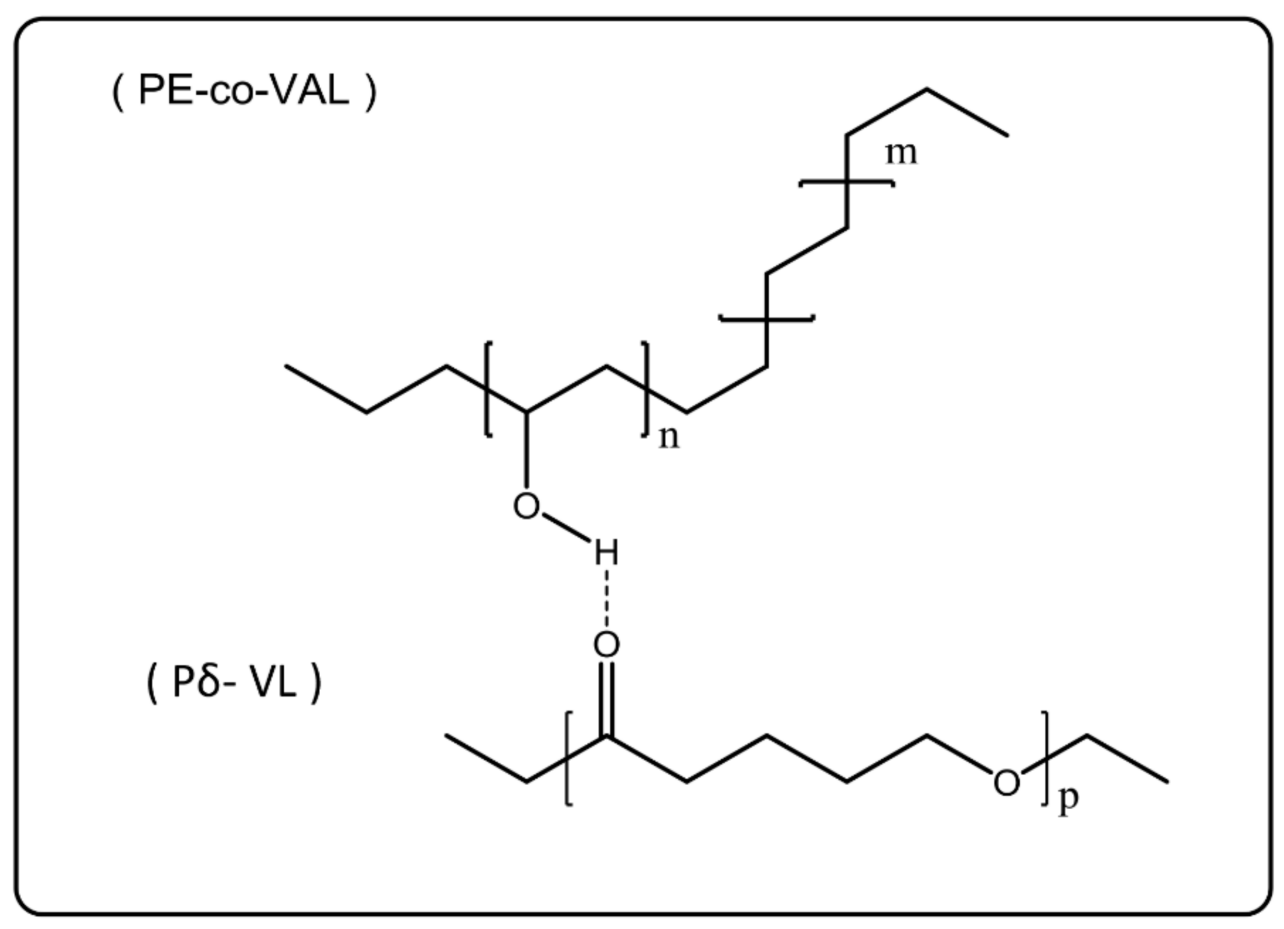
Hydrogen bond interactions between hydroxyl groups of PE-*co*-VAL and the carbonyl groups of PDVL.

A comparison between the FTIR spectra of ASA/PE-*co*-VAL/Pδ-VL50 composites with those of their components, shown in Figure 2, reveals a small shift toward the low wave numbers and an enlargement of the absorption band assigned to the carbonyl group of both ASP and δ-VL units of Pδ-VL localized at 1705 cm^−1^. These facts are without doubt attributed to a dynamic caused by the hydrogen bond interactions between the hydroxyl groups of PE-*co*-VAL and the carbonyl groups of ASP (Scheme 1) and between hydroxyl groups of PE-*co*-VAL and the carbonyl groups of PDVL (Scheme 2). This interactional dynamic is also confirmed in these same spectra by the broad band localized between 3200 and 3700 cm^−1^ overlapping the absorption band of the hydroxyl groups of PE-*co*-VA at 3475 cm^−1^ and that of the carbonyl ester groups of ASP at 3345 cm^−1^ and others bands owing to hydrogen bonds developed between them as presented in Scheme 1c. This finding also indicates that ASP is uniformly and molecularly dispersed in the PE-*co*-VAL/Pδ-VL matrix and supports our justification regarding the increase in cell adhesion on the polymeric material prepared.

### 3.3. DSC Analysis

**Figure 3:**
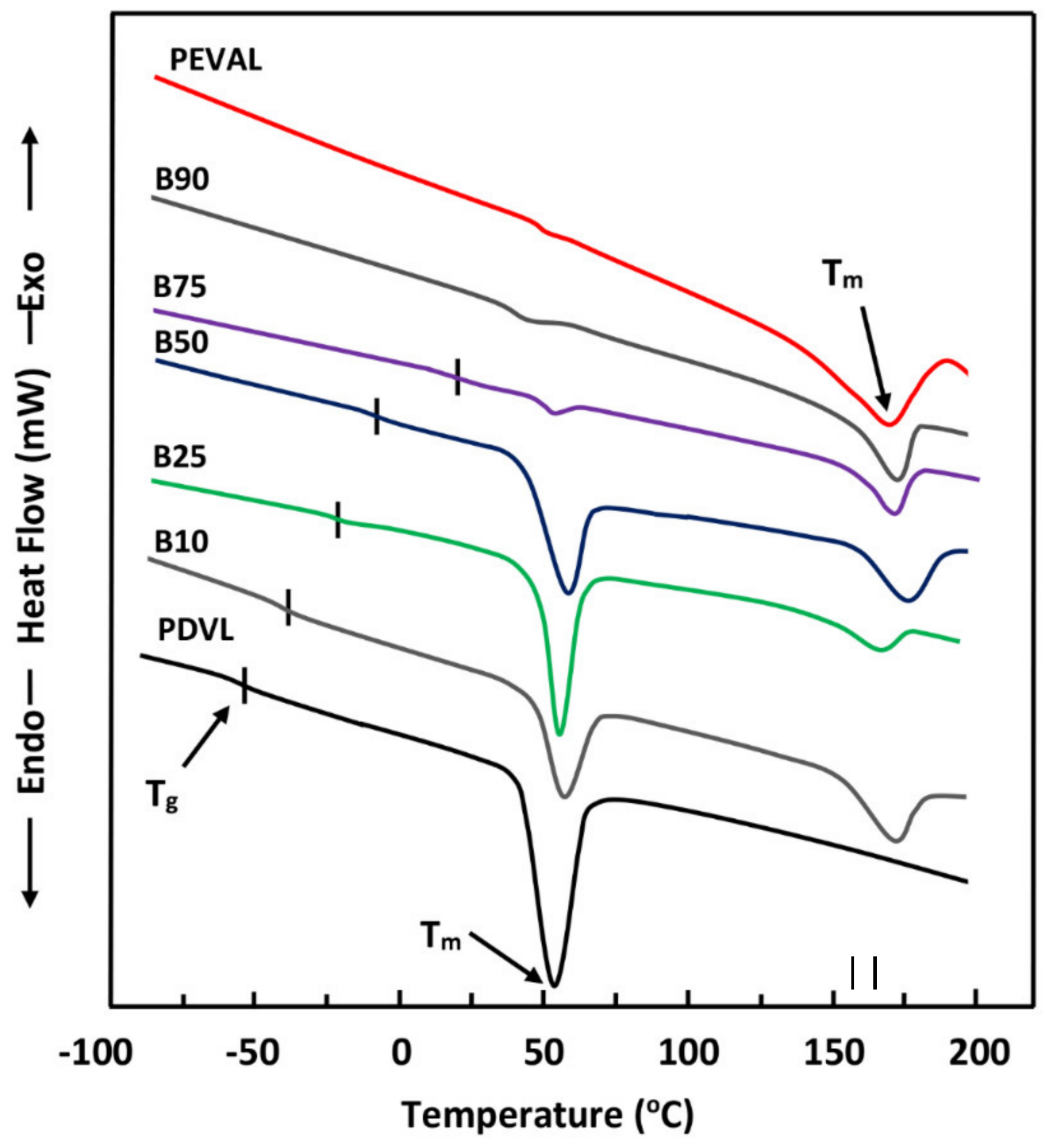
DSC thermograms of pure PE-*co*-VAL, pure Pδ-VL, and PE-*co*-VAL/Pδ-VL blends containing 90 wt% of PE-*co*-VAL (B90), 75 wt% of PE-*co*-VAL(B75), 50 wt% of PE-*co*-VA(B50), 25 wt% of PE-*co*-VAL (B25), and 10 wt% contents.

**Figure 4:**
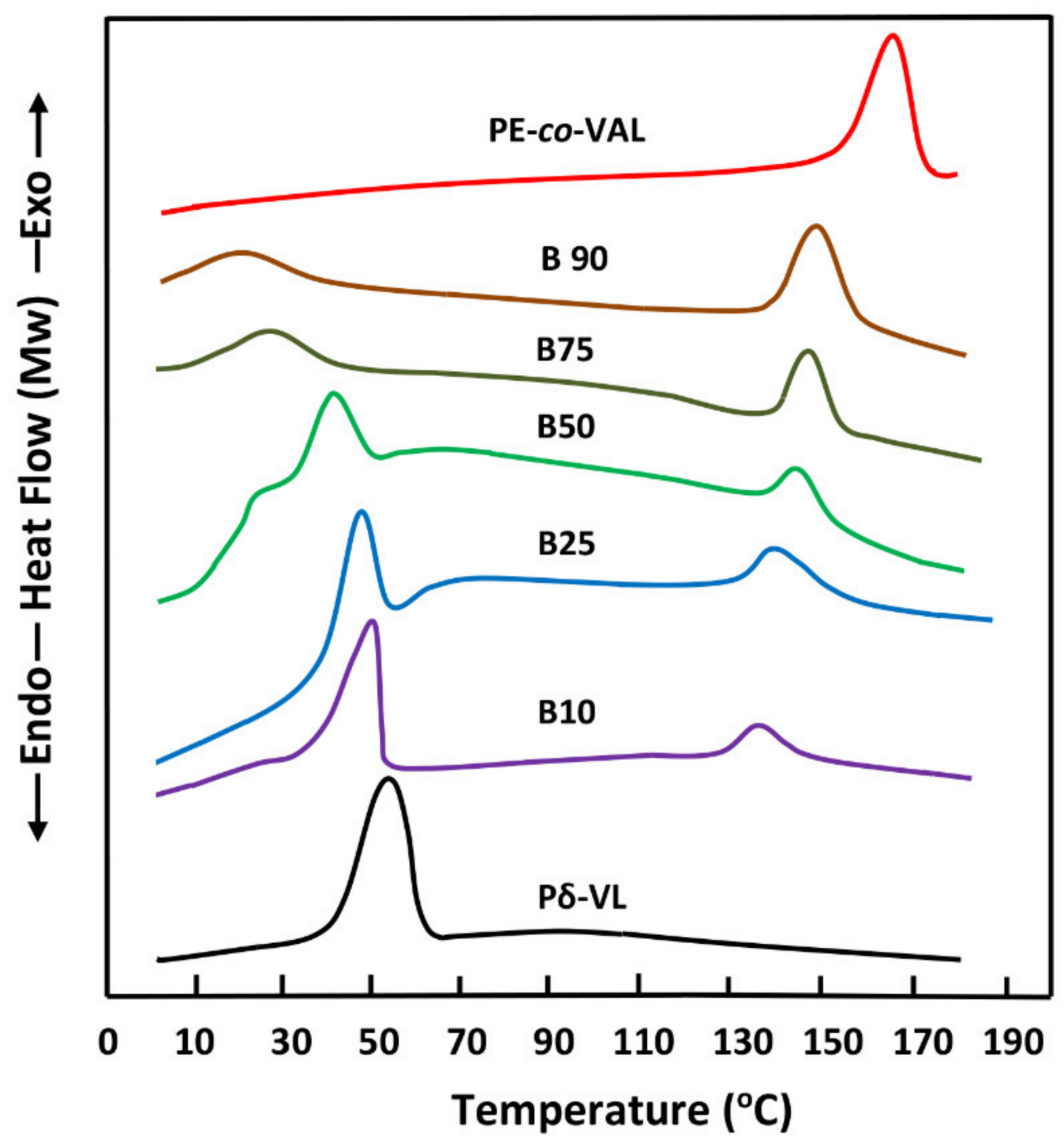
The DSC thermograms of blends and their pure components performed in the cooling mode during the non-isothermal crystallization process

**Figure 5:**
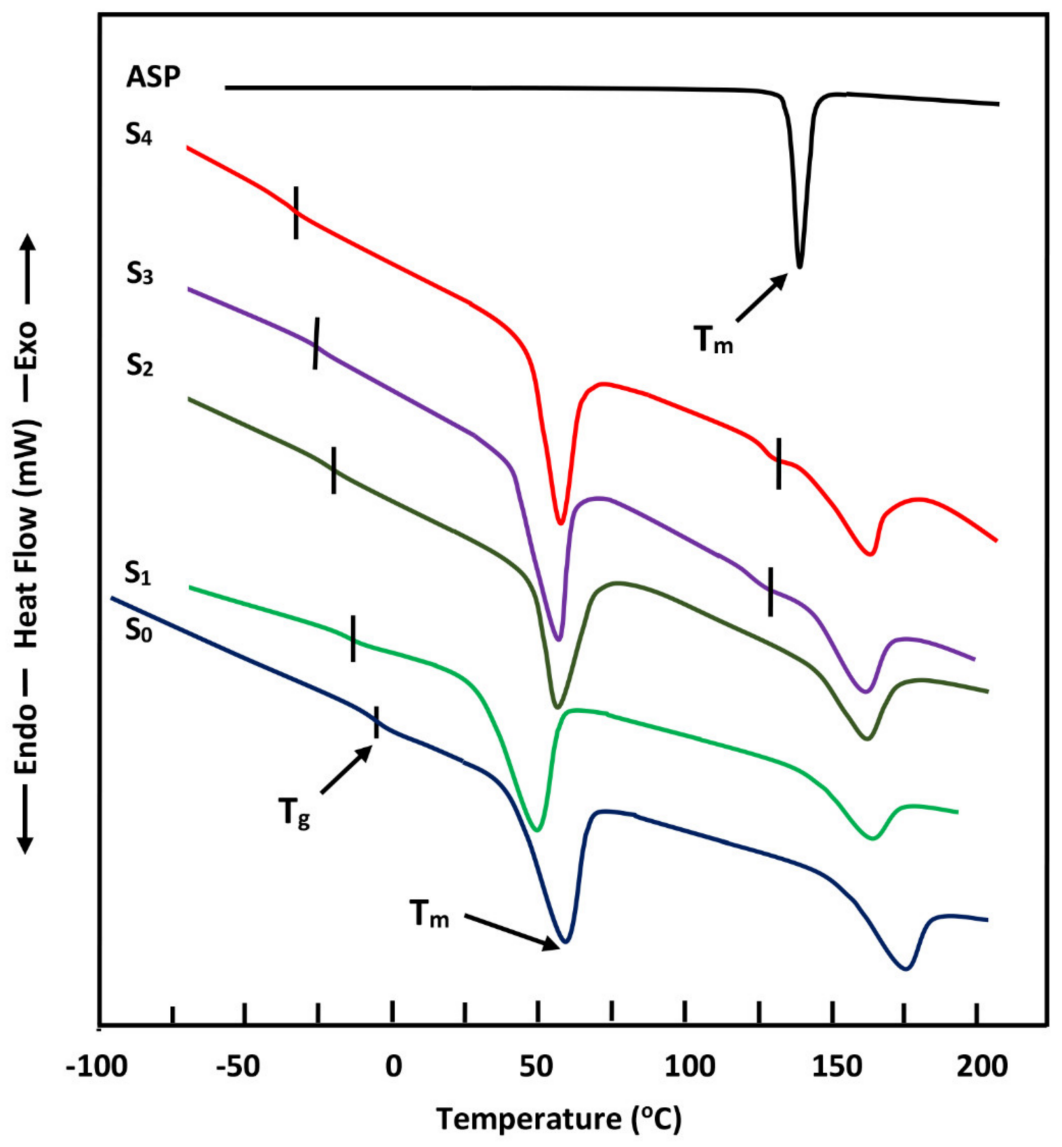
DSC thermograms of: ASP, PE-*co*-VAL/PδVL50 and ASP/PE-*co*-VAL/Pδ-VL50 containing 2wt% of ASP (S_1_), 5 wt% of ASP(S_2_), 7 wt% of ASP(S_3_), and 10 wt% of ASP (S_4_).

The DSC analysis is used in this work to examine the homogeneity of the drug-carrier system. The DSC thermograms of PE-*co*-VAL/Pδ-VL blends and those of their components are shown in Figure 3 and the data deducted are grouped in Table 3. These reveal, for all compositions, only a single *T_g_*, localized between those of the pure components and prove the total miscibility of this pair of polymers in amorphous portions. According to Qui et al. [36,38] the appearance of a single *T_g_* in the DSC thermogram for a blend is an indication of a full miscibility in the 20–40 nm scale. The shift in the melting point of PE-*co*-VAL toward the lower temperatures observed on all thermograms of the blends is probably because of the fusion of ‘Pδ-VL, which occurs at 53 °C. In this condition, Pδ-VL, being in its liquid state, plays the role of a softener for PE-*co*-VAL, thereby decreasing its *T*_m_ value. The DSC thermograms of blends and their pure components performed in the cooling mode during the non-isothermal crystallization process are gathered in Figure 4 and the data collected are added in Table 3. The curve profiles of these thermal patterns exhibit two exothermic peaks attributed to the crystallization of PE-*co*-VAL and Pδ-VL in the blends. The variation in the normalized crystallinity of the blends with the PE-*co*-VAL component reveals a shift toward low temperatures, accompanied by a drastic decrease in the crystallinity peak of PE-*co*-VAL with increasing Pδ-VL loading. As can be seen on these thermograms, the incorporation of this copolymer in the Pδ-VL matrix severely reduces their respective crystallinities. The DSC thermograms of ASP/PE-*co*-VAL/Pδ-VL50 composites containing different ASP contents are shown in Figure 5. These thermal curves also show an ultimate *T_g_* for each composite, which indicates that the miscibility of this pair of polymer was not affected by the incorporation of ASP into the polymer matrix in this composition range. However, a shift in the *T_g_* value of the blend toward the lowest temperatures is observed, which confirms the uniform distribution of the ASP in its molecular state in the polymer matrix. The shift toward the lowest temperatures is principally caused by the presence of ASP molecules encrusted in the polymer matrix, acting as wheels, promoting the chain sliding. Similar observations were also revealed by different authors working on polymer composites [39,40,41,42]. A supplementary transition is also observed at 135 °C on the thermograms of the composites containing 3 and 4 wt% of ASP contents attributed to the melting temperature of ASP particles, thereby indicating the presence of a part of ASP aggregated and uniformly dispersed in the polymer matrix.

### 3.4. XRD Analysis

**Figure 6:**
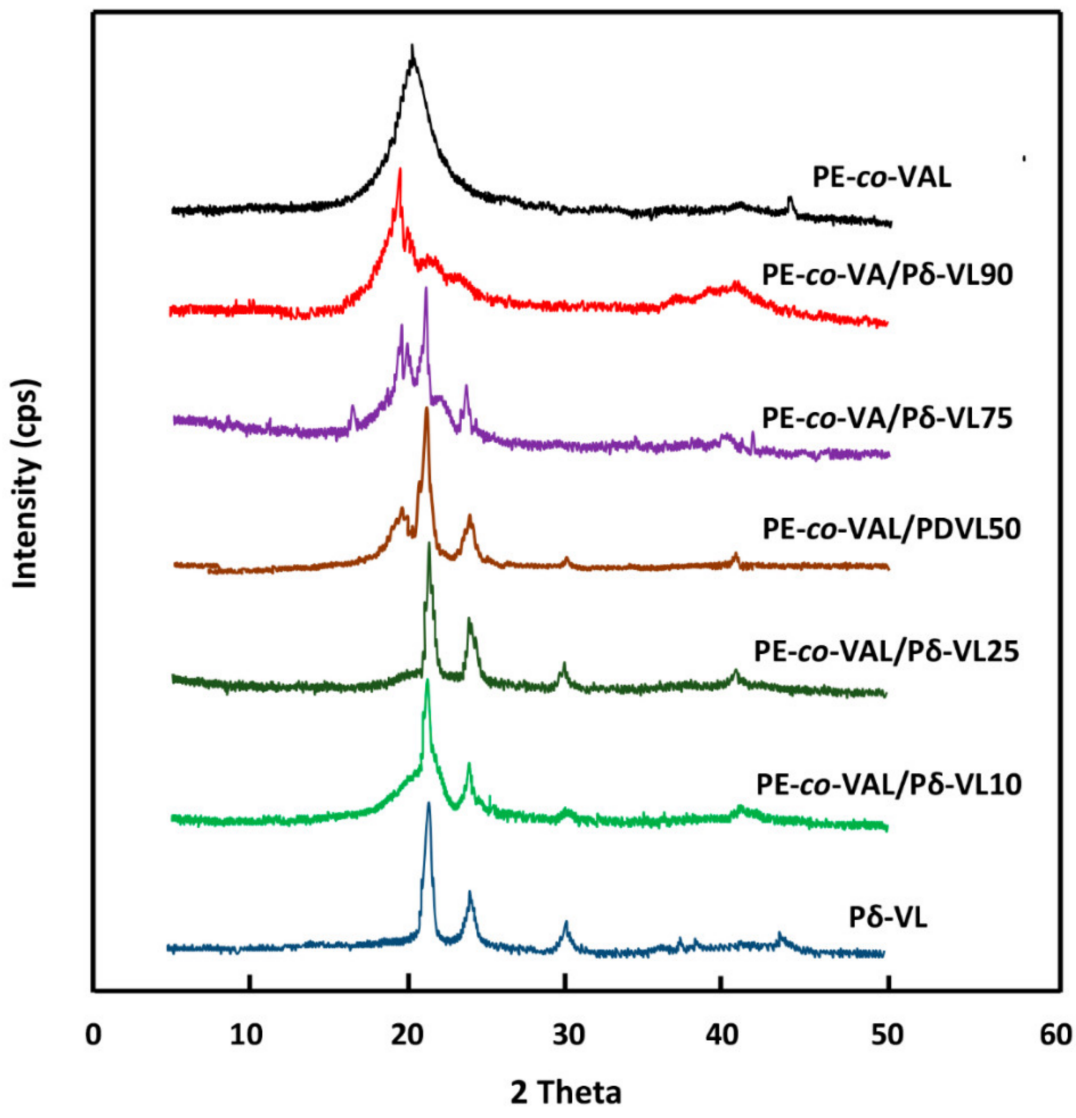
XRD patterns of PE-*co*-VAL, Pδ-VL, and PE-*co*-VAL/Pδ-VL blends with different compositions.

**Figure 7:**
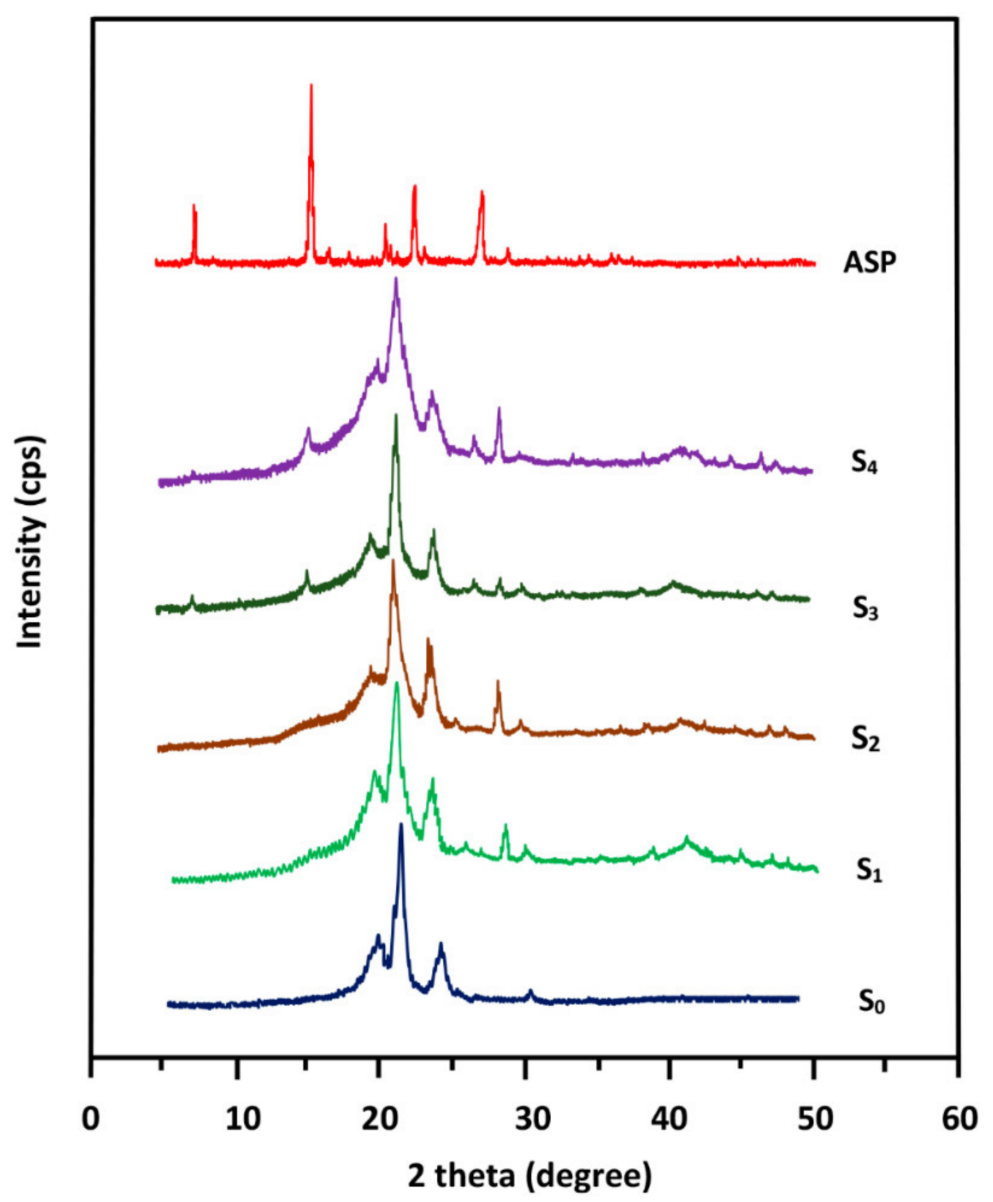
XRD patterns of PE-*co*-VAL/Pδ-VL50 and ASP/PE-*co*-VAL/Pδ-VL composites with different ASP loads: PE-*co*-VAL/Pδ-VL (S_0_); ASP-2/PE-*co*-VAL/Pδ-VL (S_1_); ASP-5/PE-*co*-VAL/Pδ-VL (S_2_); ASP-7/PE-*co*-VAL/Pδ-VL (S_3_), and ASP-10/PE-*co*-VAL/Pδ-VL (S_4_).

XRD analysis has been used in this work to confirm the results obtained by DSC regarding the uniform dispersion of ASP in the polymer matrix in its molecular state. The XRD patterns of PE-*co*-VAL/Pδ-VL blends containing different Pδ-VL contents and their pure components are presented in Figure 6. As can be seen in the Pδ-VL spectrum, three principal peaks are localized at 21.2°, 23.7°, and 30.0° 2θ characterizing the semi-crystalline structure of this polymer, while that of the PE-*co*-VAL presents only an ultimate, intense, and broad signal centered at 2θ = 20.03 attributed to the crystalline fraction of this copolymer. However, the comparison of the XRD patterns of PE-*co*-VAL/Pδ-VL blends with those of the pure constituents reveals practically the same absorption behavior by both the polymers shown. This indicates the presence of two distinct crystalline regions—one rich in PE-*co*-VAL and the other rich in Pδ-VL. However, the miscibility of this pair of polymers was realized only in their amorphous phases. Based on the DSC results and XRD analysis, it can be concluded that the components of this system crystallize separately in the blend. The comparison of the XRD patterns of ASP/PE-*co*-VAL/Pδ-VL50 composites with those of their components is shown in Figure 7. The ASP pattern reveals five main absorption peaks localized at 7.7°, 15.6°,20.6°, 22.6°, and 27.08° 2θ, corresponding to the characteristic crystal planes of ASP (100, 002, 012, 211, 310) [43]. The ASP/PE-*co*-VAL/Pδ-VL50 spectra yielded the same five peaks as of the PE-*co*-VAL/Pδ-VL50 blend at 20°, 21°, 24°, 30°, and 41° 2θ and those of the pure ASP, except those containing a relatively high ASP content (ASP-7/PE-*co*-VA/Pδ-VL50 and ASP-10/PE-*co*-VAL/Pδ-VL50), in which their XRD patterns show a total disappearance of the ASP traces. The appearance of the absorption peaks in the XRD patterns of the composites indicates a uniform dispersion of ASP in its aggregate form; whereas their disappearance reveals a uniform dispersion of this pharmacon in its molecular state.

### 3.5. Swelling Behavior

Another important factor that matters in the drug delivery process is the time it takes for a drug to release. In general, the more the reduction in swelling, the less is the water in the polymer carrier, less is the drug amount dissolved in the hydrogel, slow is the release rate, and long is the drug release time. The capacity of PE-*co*-VAL/Pδ-VL hydrogels and their pure components to swell in water was evaluated at 37 °C in media with pH values of 1, 3, 5, and 7. The maximum swellings of all specimens are given in Table 4. As can be seen from the table, for the pure PE-*co*-VAL, the swelling at saturation S_∞_ corresponding to the swelling maximum is reached in approximately 6 h of the swelling process. During this period, the maximum swellings are obtained in media with pH values of 1 and 7 when 8.31and 9.10 wt% of water was absorbed, respectively. As can be also seen from these data, owing to the hydrophobic character of Pδ-VL, its addition to the PE-*co*-VAL in an equal ratio reduces the latter’s capacity to swell by approximately half in media with pH values of 1 and 7 at 37 °C.

### 3.6. Surface Morphology of the PE-co-VAL/Pδ-VL Blend

**Figure 8:**
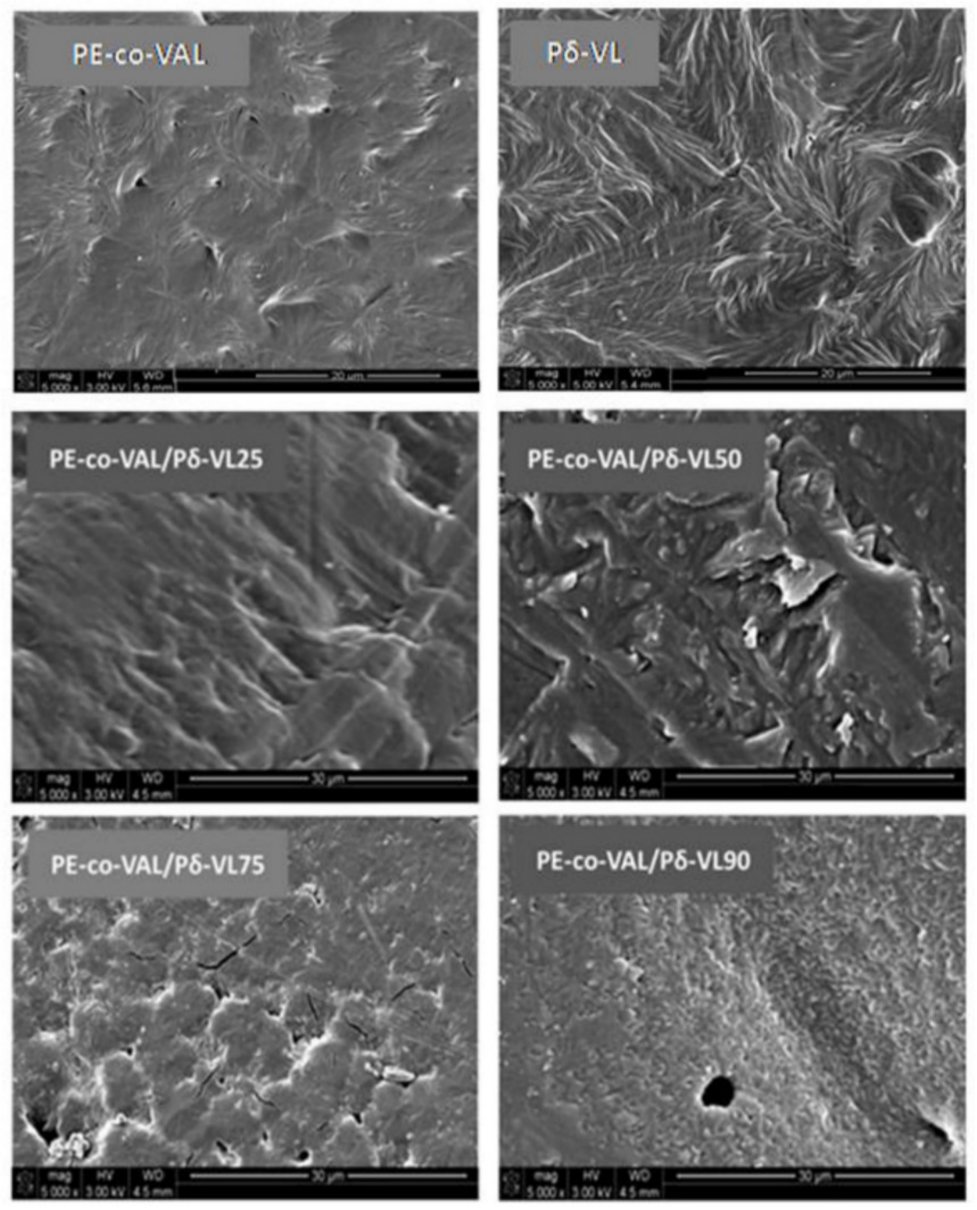
SEM micrographs of the surface morphologies of PE-*co*-VAL/Pδ-VL blends with different compositions.

The SEM micrographs in Figure 8 show surface morphologies of pure PE-*co*-VAL, pure Pδ-VL, and the PE-*co*-VAL/Pδ-VL blends of different compositions. As can be seen from the images of pure constituents, the smooth and frosted morphology surface was probably caused during vacuum drying of the samples, indicating swelling has occurred under the effect of evaporation of the residual solvent then an explosion leaving traces resembling a parachute after use. The micrographs of blends show surfaces devoid of any distinguishable zones or stress prove the miscibility of this pair of polymers in the composition range studied. Note that the mixture containing more than 50 wt% of Pδ-VL, as shown in the image of the PE-*co*-VAL/Pδ-VL90 blend (bottom right), has a porous or rough surface morphology, probably produced during the evaporation of the solvent while preparing the blend, which is favored by a superior *T*_g_.

### 3.7. Mechanical Properties

**Figure 9:**
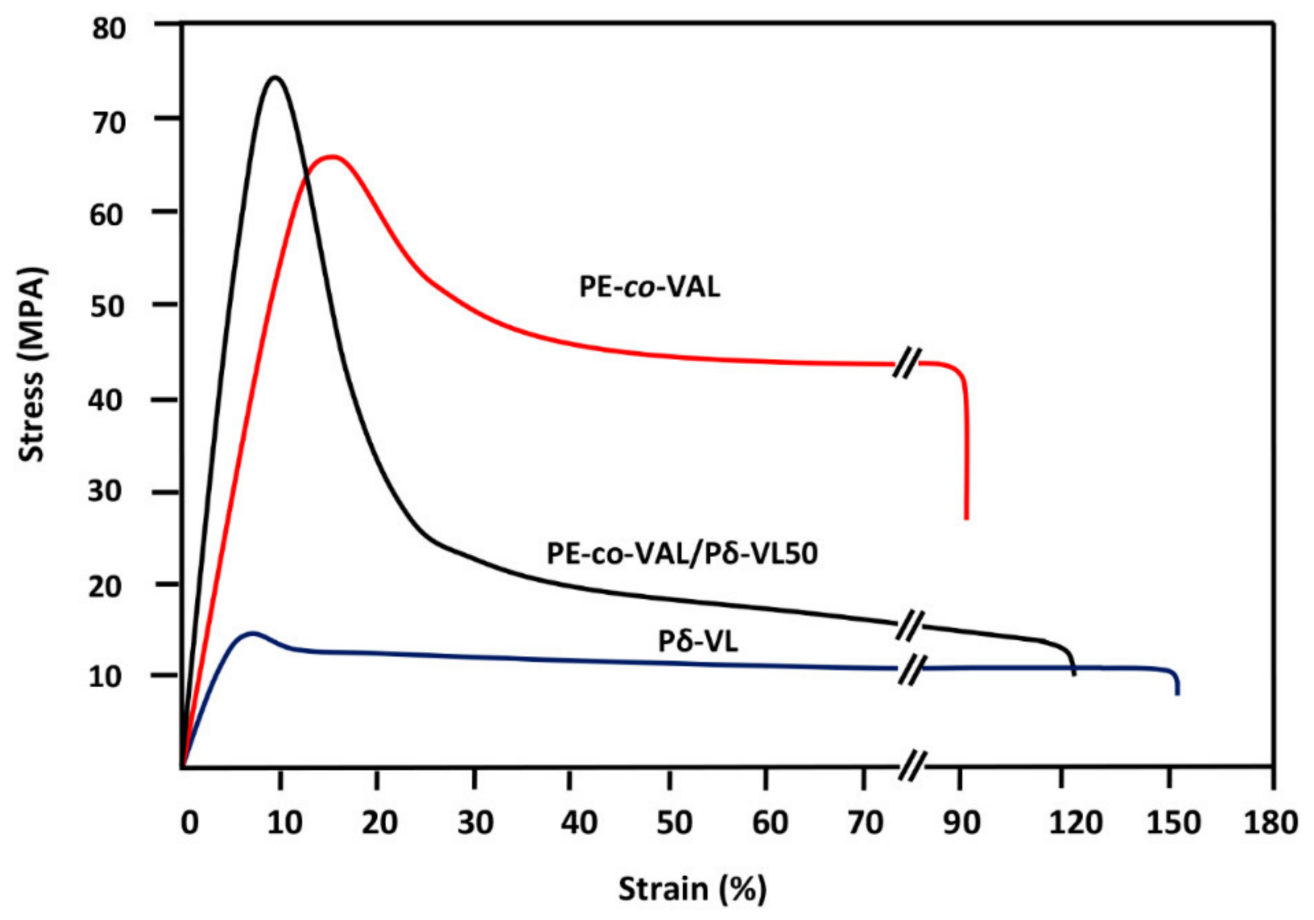
Strain-stress curves of PE-*co*-VAL/Pδ-VL50 system and their components.

Since mechanical properties of a material used in the biomedical field, notably in the field of drug delivery, play a very important role, the study of these properties is necessary. The pure PE-*co*-VAL film specimen has a mechanical consistency that allows handling and bending of the film at an angle of 180°without breaking. The PE-*co*-VAL/Pδ-VL50 specimen was subjected to a stress–strain test at 0.01 s^−1^ strain rate to understand the effect of Pδ-VL, incorporated in the PE-*co*-VAL matrix, on the modulus and mechanical properties of the blend. Figure 9 shows the stress–strain curves for the PE-*co*-VAL/Pδ-VL film specimen and those of their pure components. Typical stress-strain curves were obtained for all specimens, and the data deducted are given in Table 5. For the pure Pδ-VL, the yield point was localized at 7.5% elongation and 14.2 MPa, closely approximating values observed by Aubin et al. [44], which were 6% and 12.5 MPa, respectively. For larger elongations, the neck and strain-strain pattern show a plateau at a stress level of 11.2 MPa to the breaking point, which occurs at elongations at 152%. Young’s modulus of pure Pδ-VL was found to be 650 MPa. The results reported by these same authors on mechanical recovery experiments revealed that Pδ-VL did not undergo permanent deformation at elongations of up to 2.2%. However, permanent deformations of 10% and 15% were observed for elongations of 4.0% and 5.0%, respectively. Here, it should be noted that a larger initial deformation leads to a significant amount of permanent deformation. The data values obtained for the pure PE-*co*-VAL reveal the yield point at 24.5% elongation and 66.5 MPa. The elongation at the break point is lower than that of pure Pδ-VL, with a value of 92.2%. On the other hand, this copolymer has a modulus comparable to that of Pδ-VL, with a value of 620 MPa. The data of the PE-*co*-VAL/Pδ-VL50 blend material show that this sample shows a clearly recognizable yield point at this composition; in addition, the tensile strength of this blend (46.6) shifted slightly toward the lower values compared to that of the virgin PE-*co*-VAL (47.2) and suddenly to higher values compared to that of the virgin Pδ-VL (9.6). At the end of this study, we can add this characteristic, which is an easy control on the mechanical properties of the PE-*co*-VAL/Pδ-VL blend, to the already proven set of biomedically desirable properties of this material.

### 3.8. In Vitro Release Dynamic

**Figure 10:**
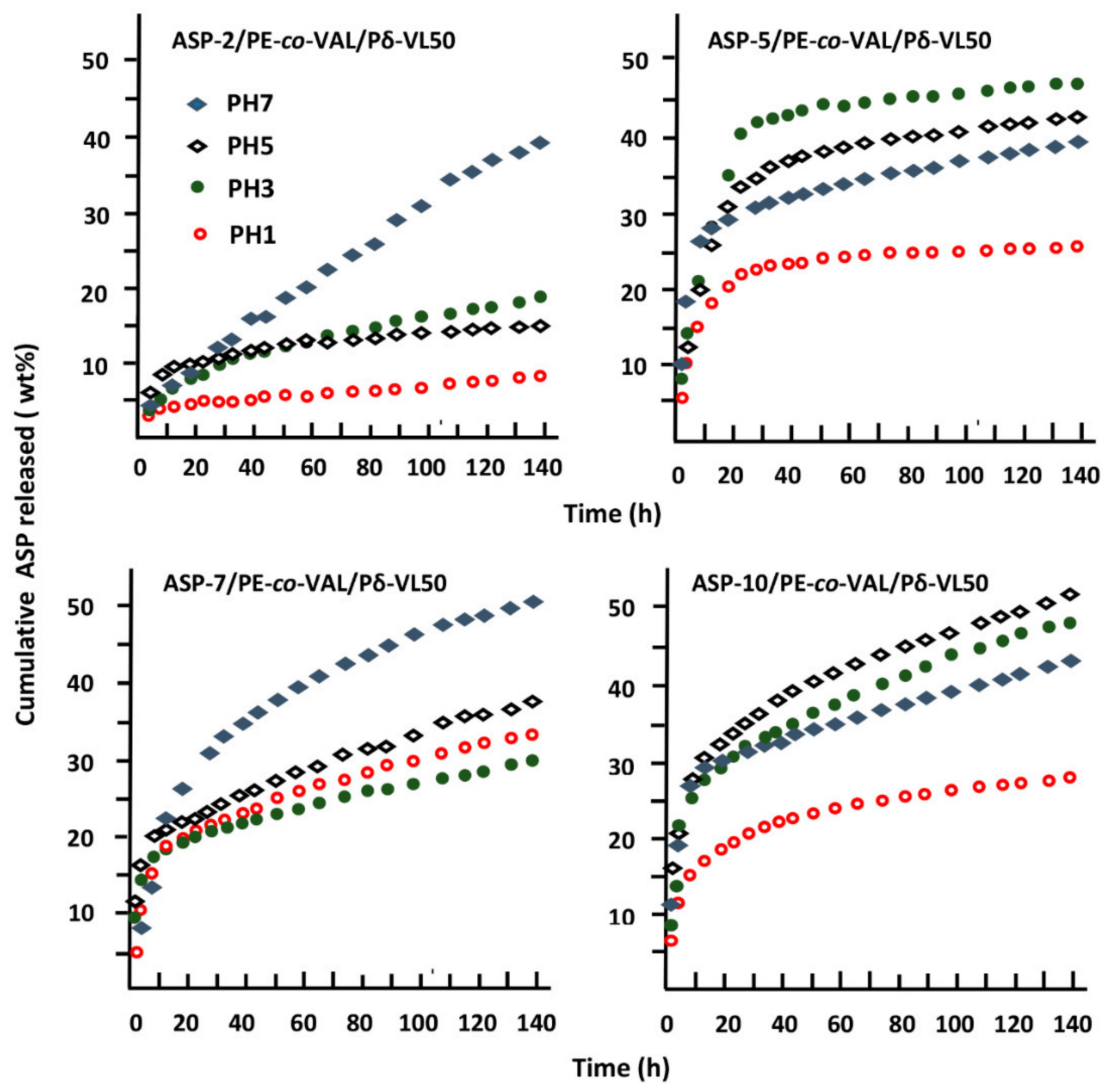
Release dynamic of ASP from the ASP/PE-*co*-VAL/Pδ-VL50 drug-carrier systems versus time.

The release dynamics of ASP from the PE-*co*-VAL/Pδ-VL drug-carrier systems evaluated through the cumulative ASP amount released in media with pH values of 1, 3, 5, and 7 at different time intervals are illustrated in Figure 10. According to these curve profiles traced, between 8 and 52 wt% of ASP was released during the release process (spanning over 6 days) depending on the initial drug amount loaded into the polymer matrix and the pH of the medium. The release dynamics indicate that, depending on the ASP load and the pH of the medium, between 20 and 75 wt% of ASP was released during the first day itself of the process. In general, the maximum ASP amount released in a neutral pH medium is observed with S_3_, in which 50 wt% from the total amount is released. However, during the same period, the minimum is reached with S_1_ in the media with pH value 1, in which the specimen was able to release only 8 wt% of the drug from its initial amount. The irregularity in the release dynamics can be principally attributed to different parameters, the main ones being the solubility of ASP in the medium inside the polymer matrix and ASP–polymer and polymer–polymer interactions. For example, the lowest release in the pH 1 medium observed for the all samples is probably because of the reduction in the hydrophilicity of PE-*co*-VAL/Pδ-VL caused by a probable esterification reaction between the alcoholic units of PE-*co*-VAL and the carboxylic acid groups of ASP. This type of reaction is usually favored in acidic media and leads to the production of new hydrophobic (ester) units insoluble in water.

#### 3.8.1. Surface Morphology of ASP/PE-*co*-VAL/Pδ-VL50 Drug-Carrier System

**Figure 11:**
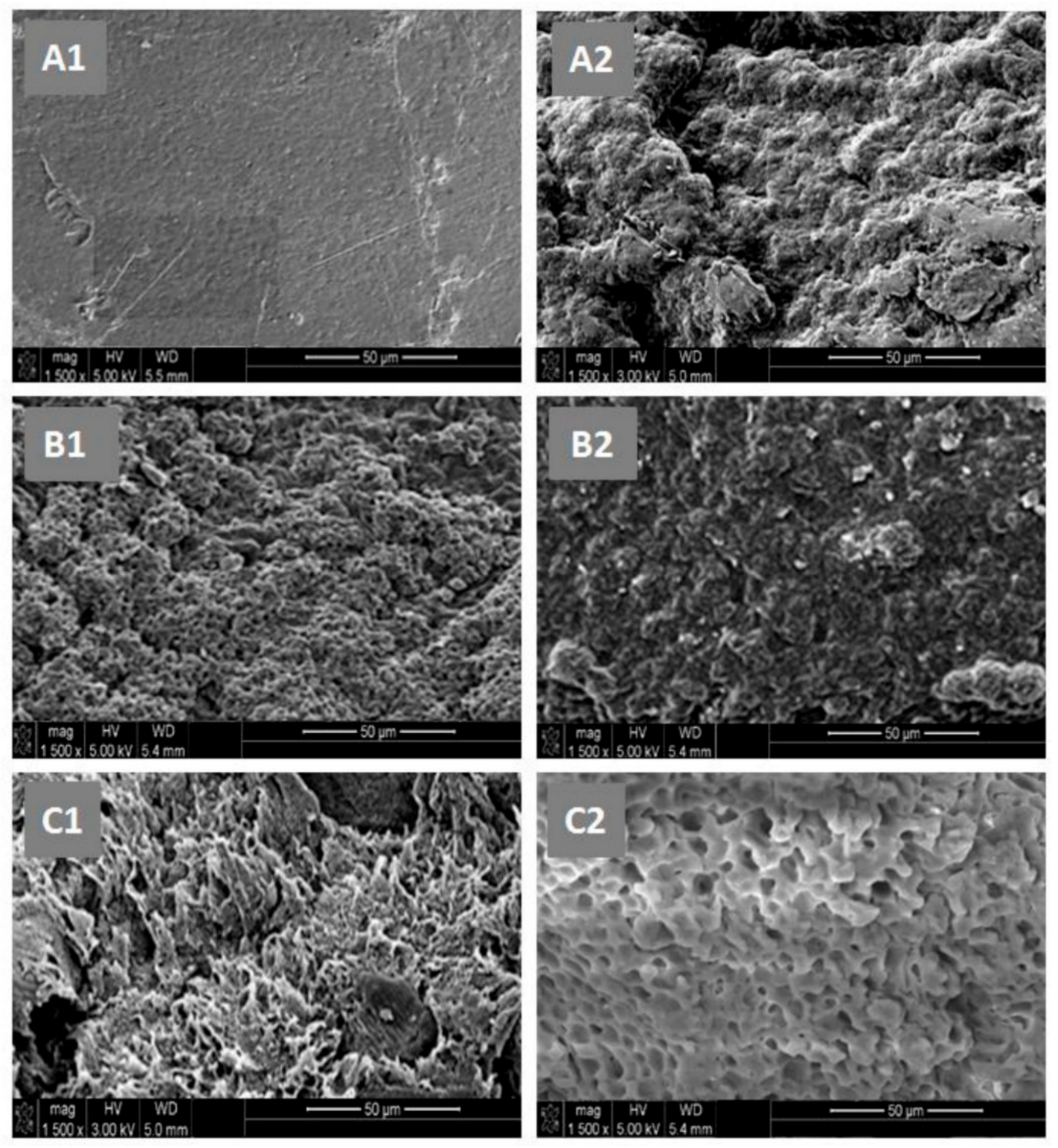
SEM micrographs of the surface morphologies of ASP-2/PE-*co*-VAL/Pδ-VL50 system (**left**) and ASP-7/PE-*co*-VAL/Pδ-VL75 system (**right**) before (**A1** and **A2**) and after the release process in neutral media pH (**B1** and **B2**) and in media pH 1 (**C1** and **C2**).

Figure 11 shows the SEM micrographs of the surface morphologies of ASP-2/PE-*co*-VAL/Pδ-VL50 and ASP-7/PE-*co*-VAL/Pδ-VL75 drug-carrier systems before and after the release process, which are selected according to their pertinent results obtained. For the micrographs of the ASP-2/PE-*co*-VAL/Pδ-VL50 system (left side), image A1 shows the surface morphology of the composite before the release process. This image, as expected, shows a uniform surface with ASP particles dispersed uniformly and well covered with polymer. As can be seen from the B1 image, which presents this same system after the release in a neutral pH medium, shows pores sized between 1 and 5 μm and uniformly dispersed on the surface, undoubtedly left slowly by the ASP particles after the release process. On the other hand, the surface morphology of the same drug-carrier system after the release of ASP in the pH 1 medium (B2 image) presents a scrubbed surface, confirming an explosive liberation of a large amount of ASP. For the ASP-7/PE-*co*-VAL/Pδ-VL75 micrographs (right side), which contains 7 wt% of ASP and 75 wt% of Pδ-VL, before the release process (image A2) shows a dense, rough surface of drug/polymer composite formed during the solvent evaporation process under vacuum. After the release of ASP in a neutral pH medium (Image B2), this system, as is the case with the ASP-2/PE-*co*-VAL/Pδ-VL50, also presents a porous surface of practically the same pore sizes but less apparent, thereby indicating a less important release dynamic. The micrograph of the surface morphology of this system, taken at the end of the release process carried out in the pH 1 medium (Image C2), shows cavities or micropores having dimensions relatively larger than those realized in the neutral pH medium, which indicates a significant amount of ASP released during the process.

#### 3.8.2. Diffusion Behavior of ASP

**Figure 12:**
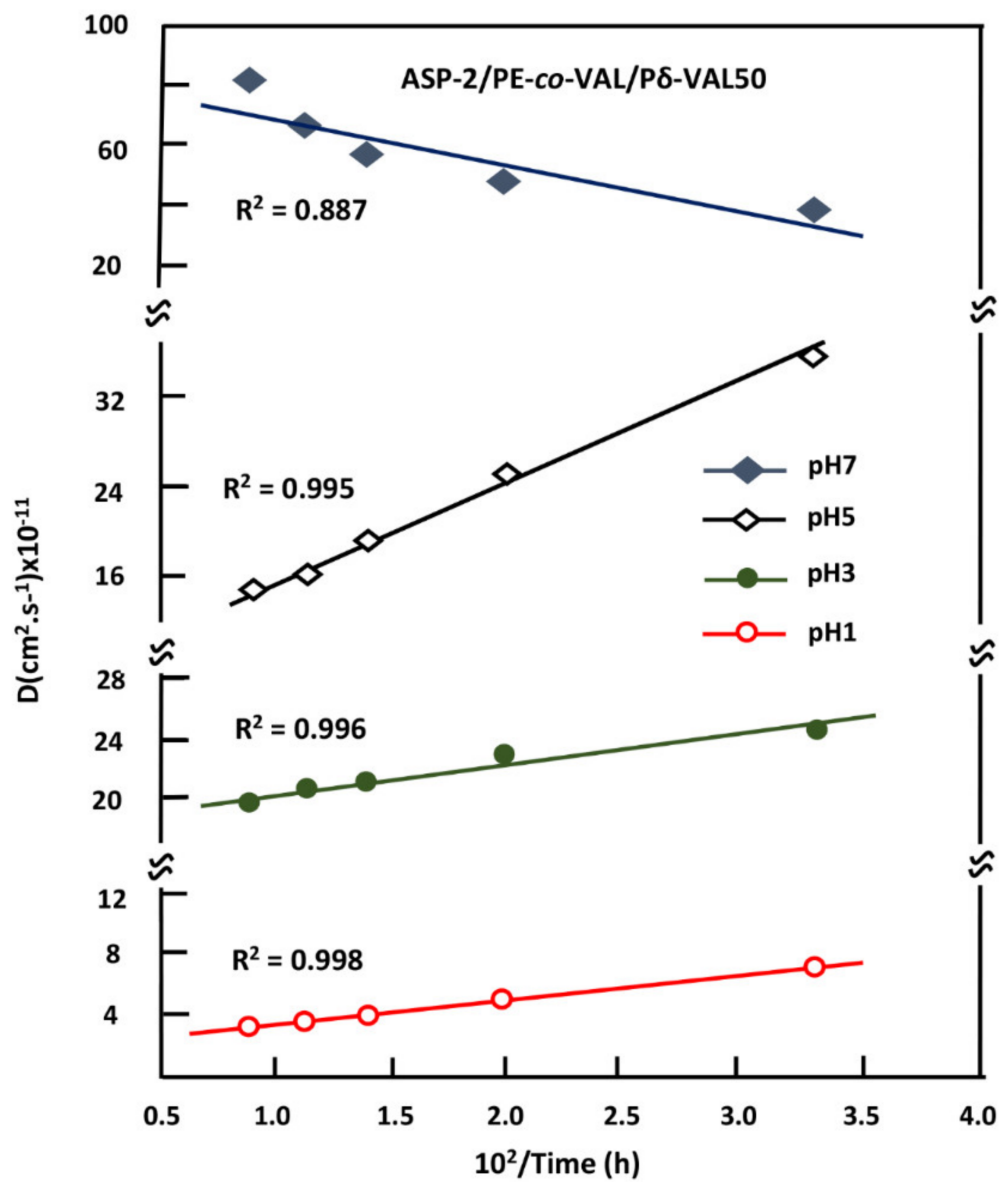
Variation of the diffusion coefficient of ASP through the PE-*co*-VAL/Pδ-VL carrier versus the inverse of temperature.

According to Lin et al. [45], the diffusion D of a drug through a polymeric material can be governed by the Fick model when the total drug released by a drug-carrier system in a medium does not exceed 60 wt% of the total drug loaded in the polymer matrix. This condition is represented by Equation (3). In this condition, the value of D is given by the following relationship [46,47,48,49]:

(3) $$D=\frac{0.196\times d^{2}}{t}{\left[\frac{m_{t}}{m_{o}}\right]}^{2}$$

where d is the thickness of the film sample, and m_o_ and m_t_ are the masses of ASP initially incorporated in the PE-*co*-VAL/PDVL50 system and that released at time t of the release process, respectively. D values are determined when the permanent regime is reached and, therefore, all ASP particles deposited or pressed on the film surface are totally removed by washing in the medium during the initial hours of the release process. Under these conditions, the curve profiles of D versus time are important and reflect exactly the dynamics of ASP released in the medium inside the polymer matrix. For the ASP/PE-*co*-VAL/Pδ-VL50 drug-carrier system, which is chosen from the previous studies as the most efficient, the variation in D versus the inverse of time calculated from the experimental data of the cumulative ASP released plotted in Figure 10 and Equation (2) are shown in Figure 12. As shown, these curve profiles of positive straight lines were obtained for each drug-carrier composition and in any pH media, except that initially containing 1.0 wt% of ASP in the pH 7 media, in which case an exponential curve is obtained. This finding indicates that the diffusion of ASP through PE-*co*-VAL/PDVL50 material obeys the Fickian model except for that containing the lowest drug content in the neutral pH media. It also indicates that the permanent regime of the dynamic release of ASP was reached. Based on these results, it was possible to shift our investigation to the second zone of the release process, which was generally localized between 20 and 140 h.

#### 3.8.3. Effect of the Initial Amount of ASP

**Figure 13:**
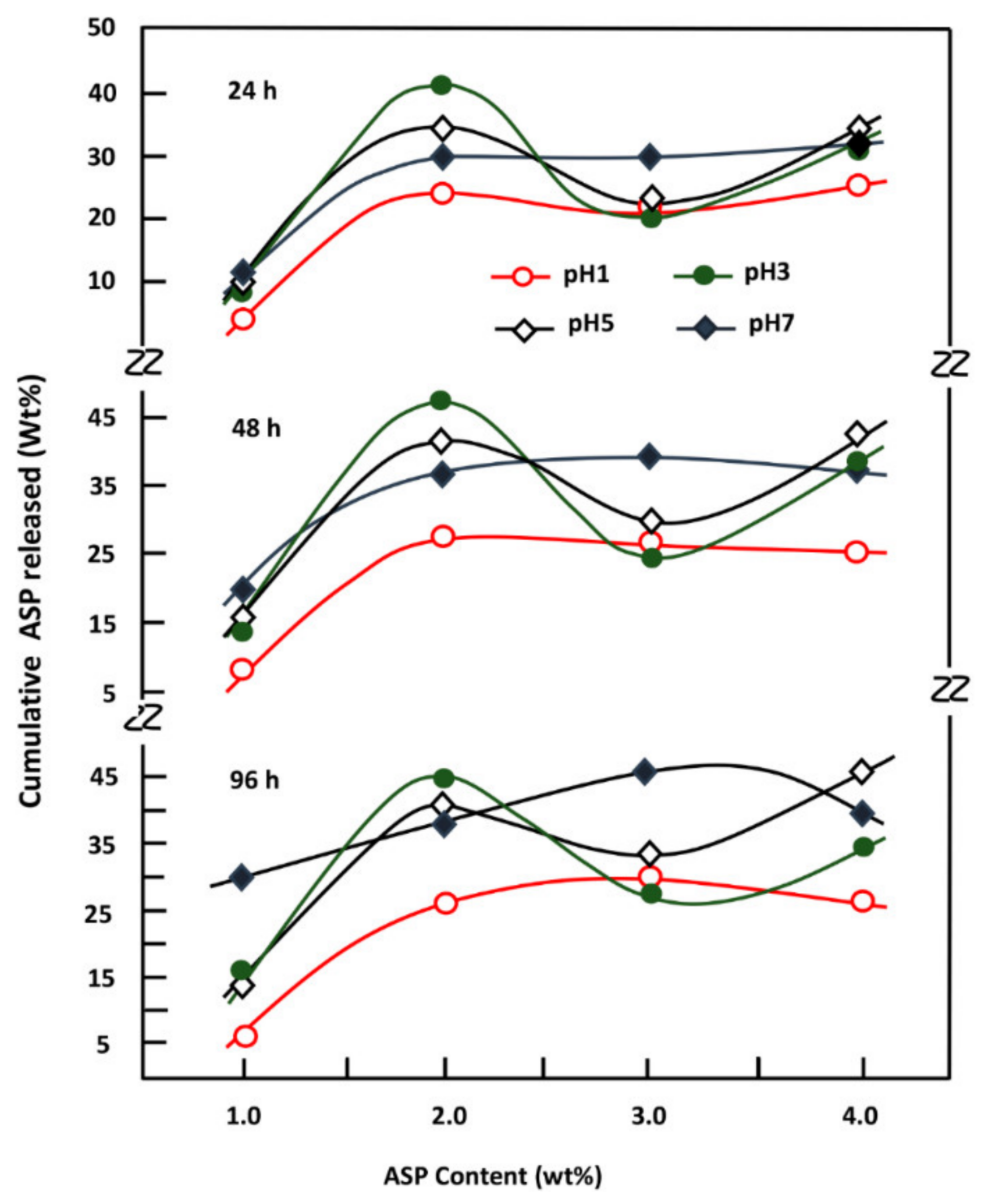
Variation of the cumulative ASP released from the ASP-2/PE-*co*-VAL/Pδ-VL drug carrier system in different media pHs versus the ASP content and at different periods.

Figure 13 shows the influence of the ASP amount loaded in the PE-*co*-VA/Pδ-VL system on the release dynamics of the drug in different pH media over 24, 48, and 96 h of the release process. As can be seen from these curve profiles, the release dynamics varied but followed the same trend for all durations. For the specimens examined in pH 1 and 7 media, the cumulative ASP released varied with the ASP content, following a same trend that increased when the drug/polymer composition is lower than 2 or 3 wt% depending to the pH of the medium, then reached a maximum or stabilized beyond that. However, for the specimens immersed in pH 3 and 5 media, the cumulative drug released also had practically the same profile in which their curves showed a maximum at 2 wt% of ASP loaded and a minimum at 3 wt%. It is also revealed that, for all specimens, the maximal value of the cumulative ASP released coincides well with the maximum solubility of ASP in water, which ranged between 2 and 3 mg/mL at 37 °C [50] depending on the pH of the medium. In light of this finding, the increase in the release dynamics can be because of an increase in the concentration of the ASP/medium solution inside the polymer matrix; however, beyond these compositions, a fraction of the aggregated drug particles remains insoluble because of the super saturation of the solution, which explains the decrease in the release dynamics at ASP loads higher than 2 or 3 wt% depending on the pH of the medium. These results can also be explained through the values of the maximum swelling of the PE-*co*-VAL/Pδ-VL specimens obtained in different pH media shown in Table 6. The highest swelling is seen in pH 1 and 7 media with 6.18 and 5.63 wt%, respectively. On the other hand, the lowest swelling is seen in pH3 and 5 media with 4.22 and 4.03 wt% of media absorbed, respectively. Therefore, the more the water absorbed by the polymer matrix, the more is the ASP amount dissolved and more is the cumulative drug released.

#### 3.8.4. Influence of the Degree of Swelling

**Figure 14:**
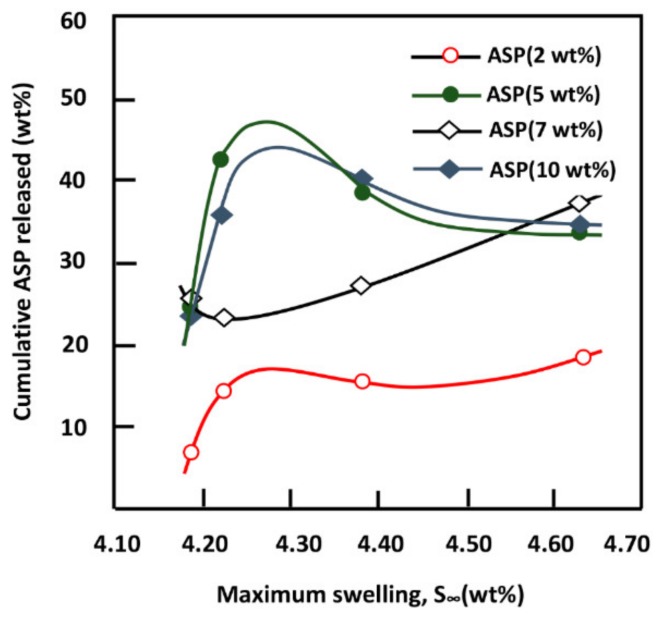
Variation of the cumulative ASP released from ASP/PE-*co*-VAL/Pδ-VL50 drug carrier systems versus the swelling degree during a period of 48 h.

The influence of the degree of swelling of the PE-*co*-VAL/Pδ-VL50 blend on the release dynamics of ASP was studied for 48 h; the results obtained are plotted in Figure 14. As shown in these curve profiles, the maximum of the cumulative ASP released is reached when the degree of swelling is 4.27, except in the case containing 7 wt% of drug, in which the release dynamics increased almost linearly.

**Figure 15:**
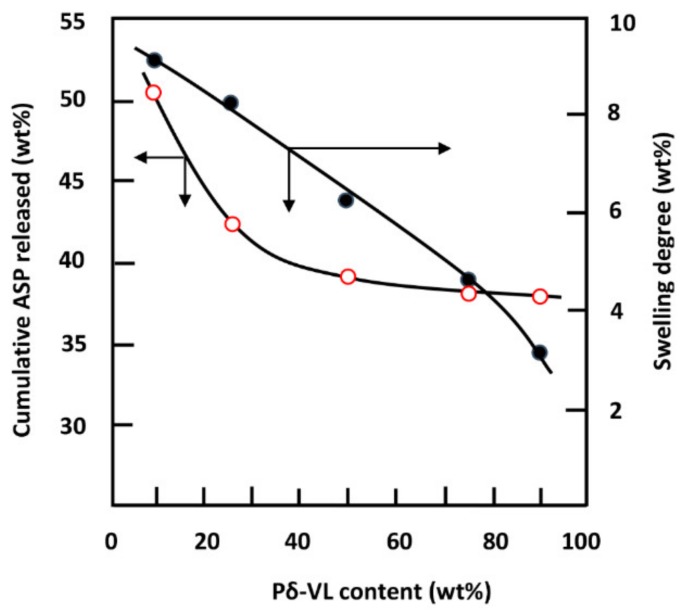
Variations of the cumulative ASP released from the ASP-7/PE-*co*-VAL/Pδ-VL drug carrier system and the swelling degree of PE-*co*-Pδ-VL in neutral pH media versus the Pδ-VL content.

The influence of the Pδ-VL content in the ASP/PE-*co*-VAL/Pδ-VL drug-carrier system initially containing 7 wt% of ASP on the release dynamics of this drug was studied in a neutral pH medium for 48 h; the results obtained are plotted in Figure 15. As shown by the obtained curve profile, the cumulative released ASP decreased suddenly as the amount of Pδ-VL increased in the range of 10–30% by weight, then continued to decrease slowly beyond. This finding seems to be obvious, because an addition of the amount of Pδ-VL (hydrophobic polymer) to the PE-*co*-VAL (hydrophilic polymer) causes a reduction in the degree of swelling of the resultant material, leading to a reduction in the amount of ASP dissolved in water inside the polymer matrix. This consequently leads, at a certain limit, to the reduction in the release dynamics. The pseudostability or the slow decrease in the release dynamics observed when the Pδ-VL content is greater than 35%, despite the degree of swelling continuing to decrease, is probably because of an additional release of an aggregated fraction of ASP remaining embedded inside micropores or on rugged surfaces created after the solvent evaporation during the drug-carrier preparation. The presence of aggregated ASP particles in the ASP/PE-*co*-VAL/Pδ-VL system was already proved through the results of the DSC, XRD, and SEM analyses in Section 3.3, Section 3.4, and Section 3.6.

#### 3.8.5. Performance of ASP/PE-*co*-VAL/Pδ-VL50 Drug-Carrier System

The kinetics of ASP release undertaken in this work was based on the data collected from the instantaneous release rates of ASP from ASP/PEL/Pδ-VL50 drug-carrier systems taken from the slopes of the linear portions of the kinetic curves presented in Figure 10. As shown in these curve profiles, two main pseudo-stable zones were obtained for all samples. The data obtained are presented in Table 6.

As can be seen from these results, it was revealed that the ASP-2/PE-*co*-VAL/Pδ-VL drug-carrier system is the most efficient, because it was able to deliver to the stomach (mediumpH1) the smallest amount of ASP (3.4 wt% over 5 h with a release rate of 0.85 wt%/h and 5.4 wt% over 135 h with a release rate of 0.04 wt%/h) and the greatest amount of ASP to the intestines (neutral pH medium) (4.0 wt% of ASP over4h with a release rate of 1.0 wt%/h and 37.0 wt% over 136 h with a release rate of 0.27 wt%/h). According to the statistics reported by Beltzer et al. [51] on the total gastrointestinal transit (GITT), it has been revealed that the average ranges between 53 and 88 h and varies according to the age, weight, and health of the person. These authors also reveal that the distribution of the transit time is spread over three main stages as follows: in stomach (1–4 h at pH of 1.5–3.5), in small intestines (4–12 h at pH of 7–9), and finally in the colon (48–72 h at pH of 5–7). Based on the data presented in Table 6 and those reported on the GITT by Beltzer et al., it was possible to estimate the distribution of the in vitro cumulative ASP released in different digestive system organs regardless of actions of any enzymes and microorganisms; the results obtained are presented in Table 7. Based on the criteria that stipulate that the best-performing drug-carrier system is the one in which the highest amount of drug is uniformly released in the intestines (neutral pH medium) over the longest time and the lowest amount of the drug is released in stomach (in acidic medium) over the shortest time. In other words, the best performance of a drug-carrier system is obtained when the ratio of the total amount of drug released in the intestines to the total amount of drug released in the stomach (intestines/stomach) is the highest. According to the data shown in Table 7, theASP-2/PE-*co*-VAL/Pδ-VL50 system containing 2 wt% of ASP seems the most efficient among the series studied, because this system has the highest intestines/stomach ratio (min = 8and max = 24). In other words, this system can deliver uniformly in the intestines between 8 and 24 times the amount of ASP in the stomach. The ASP-10/PE-*co*-VAL/Pδ-VL50 system containing 10 wt% of ASP occupies the second position with a minimum ratio of 19.98 and a maximum of 5.48.

## 4. Conclusions

This investigation is the first step to know some essential properties of a potential material such as the PE-*co*-VAL/Pδ-VL50 blend used to control the oral administration of drugs. The prepared blend was proved miscible in all compositions by using FTIR, DSC, and XRD analyses, thereby proving the uniform dispersion of each polymer in the other. The blend also has low values of *T_g_* (−33 and 25 °C), indicating that the resultant copolymer is in its soft state at the body temperature. The assessment of cell adhesion and growth revealed that the LoVo cell adherence was most visible on the blend containing an equal amount of each component. Moreover, the specific growth during both the 24 and 48 h culture periods reflected the highest growth rate on the same material. The swelling test of the prepared material showed that no traces of these two polymers are dissolved in media of different pH values but swell moderately. It was also noted that the addition of Pδ-VL to PE-*co*-VAL reduces the degree of swelling of the latter polymer. For example, at equal ratios of Pδ-VL and PE-*co*-VAL, the maximum swelling degree was reduced by about half the capacity of the PE-*co*-VAL polymer to swell. The DMA analysis revealed, in general, that the mechanical properties of the PE-*co*-VAL/Pδ-VL50 blend were localized between those of the pure components, except those of the modulus and yield at stress, which were superior. The in vitro release dynamics of ASP in different pH media from PE-*co*-VAL/PDVL50material indicates that the diffusion of this drug is governed by the Fickian model, except in the case containing the lowest drug content (2 wt%) in a neutral pH media. The effect of the ASP content in the PE-*co*-VA/Pδ-VL system on the release dynamics of this drug at different pH media revealed that, for all specimens, the maximal value of the cumulative ASP released coincides well with the maximum solubility of ASP in water depending on the pH of the medium. The influence of the degree of swelling of the PE-*co*-VAL/PDVL50 on the release dynamics of ASP indicates that, in general, the maximum of the cumulative ASP released is reached when the degree of swelling is 4.27. For a period of 48 h in a pH 7 medium, the influence of the Pδ-VL50 content in the ASP-7/PE-*co*-VAL/Pδ-VL drug-carrier system on the release dynamics revealed a sudden decrease in the cumulative ASP released when the Pδ-VL load increased between 10% and 30% by weight, then stabilized or continued to decrease slowly beyond. To conclude this work, it was found that the ASP-2/PE-*co*-VAL/Pδ-VL drug-carrier system is the most efficient. Based on the data obtained and the gastrointestinal transit time reported by Beltzer et al., it was possible to estimate the distribution of the in vitro cumulative ASP released in different digestive organs regardless of the actions of any enzymes and microorganisms and select the best-performing drug-carrier system.

## Figures and Tables

**Table 1:** Preparation conditions of PE-*co*-VAL/Pδ-VL blends.

Sample	PE-*co*-VAL (g)	PE-*co*-VAL (wt%)	Pδ-VL (g)	Pδ-VL (wt%)
PE-*co*-VAL	10.0	100	0	0
PE-*co*-VAL/Pδ-VL10	9.0	90	1.0	10
PE-*co*-VAL/Pδ-VL25	7.5	75	2.5	25
PE-*co*-VAL/Pδ-VL50	5.0	50	5.0	50
PE-*co*-VA/Pδ-VL75	2.5	25	7.5	75
PE-*co*-VA/Pδ-VL90	1.0	10	9.0	90
Pδ-VL	0	0	10.0	100

**Table 2:** Preparation conditions of ASP/PE-*co*-VAL/Pδ-VL50 drug-carrier-systems.

System Code	PE-*co*-VAL (g)	Pδ-VL (g)	ASP (g)	ASP (wt%)
ASP-2/PE-*co*-VAL/Pδ-VL50	0.5	0.5	0.020	2.0
ASP-5/PE-*co*-VAL/Pδ-VL50	0.5	0.5	0.052	5.0
ASP-7/PE-*co*-VAL/Pδ-VL50	0.5	0.5	0.075	7.0
ASP-10/PE-*co*-VAL/Pδ-VL50	0.5	0.5	0.111	10.0

**Table 3:** Variation of *T_g_*, *T_m_* and *T_c_* of the blends versus the composition.

System	PEVAL/PDVL Composition (wt%)	T_g_^Exp^ (°C)	T_m_ (°C)	T_c_ (°C)
PE-*co*-VAL	100:0	50	170	165
PE-*co*-VAL/Pδ-VL90	90:10	40	174–42	149–50
PE-*co*-VAL/PδVL75	75:25	20	172–52	148–48
PE-*co*-VAL/Pδ-VL50	50:50	–10	175–60	145–42
PE-*co*-VAL/Pδ-VL25	25:75	–22	170–58	140–27
PE-*co*-VAL/Pδ-VL10	10:90	–37	174–59	136–21
Pδ-VL	0:100	–53	53	53

**Table 4:** Data of the maximum swelling degree of PE-*co*-VAL/Pδ-VL hydrogels and their components in different media pH and at 37 °C.

System	Maximum Swelling (S_∞_) (wt%)			
	pH1	pH3	pH5	pH7
PE-*co*-VAL	8.31	6.21	6.34	9.10
PE-*co*-VAL/Pδ-VL10	7.22	5.33	5.65	8.18
PE-*co*-VA/Pδ-VL25	5.98	5.07	4.98	6.23
PE-*co*-VAL/Pδ-VL50	4.18	4.22	4.03	4.63
PE-*co*-VAL/Pδ-VL-75	3.24	3.15	3.23	3.64
PE-*co*-VA/Pδ-VL-90	3.18	2.76	2.22	3.25
Pδ-VL	2.33	2.53	0.66	1.56

**Table 5:** Mechanical properties of PE-*co*-VAL/Pδ-VL50 blend and its pure components.

Sample Code	Modulus (MPa)	Yield Stress (MPa)	Elongation at Yield (%)	Tensile Strength	Stress at Break (MPa)	Elongation at Break (%)
Pδ-VL	620	14.2	7.5	9.6	11.2	152.4
PE-*co*-VAL/Pδ-VL-50	1040	77.6	9.9	46.6	24.1	123.2
PE-*co*-VAL	651	66.5	24.5	47.2	41.3	92.2

**Table 6:** The results obtained from the main pseudo-stable zones of the dynamic release of ASP from the ASP/PE-*co*-VAL/Pδ-VL50 drug-carrier systems.

Drug-Carrier System	pH Media	Period (h)	ASP Released (wt%)	Release Rate (wt%/h)	Drug-Carrier System	pH Media	Period (h)	ASP Released (wt%)	Release Rate (wt%/h)
S1^*^	1	0–4	3.4 ± 0.3	0.85± 0.08	S2^*^	1	0–4	15.0 ± 0.5	3.75 ± 0.13
		5-140	5.0 ± 0.3	0.04 ± 0.01			22–140	04.0 ± 0.3	0.03 ± 0.01
	3	0–32	11.0 ± 0.4	0.34 ± 0.01		3	0–22	35.0 ± 0.7	1.59 ± 0.03
		32-140	18.0 ± 0.5	0.15 ± 0.01			29–140	03.5 ± 0.3	0.03 ± 0.01
	5	0–12	10.0 ± 0.4	0.83 ± 0.03		5	0–12	15.0 ± 0.5	1.25 ± 0.04
		12-140	5.0 ± 0.3	0.04 ± 0.01			32–140	06.0 ± 0.3	0.06 ± 0.01
	7	0–4	4.0 ± 0.3	1.00 ± 0.08		7	0–5	26.0 ± 0.6	5.20 ± 0.12
		4–140	37.0 ± 0.7	0.27± 0.01			18–140	10.0 ± 0.5	0.08 ± 0.01
S3^*^	1	0–5	15.0 ± 0.6	3.00± 0.12	S4^*^	1	0–6	12.0 ± 0.6	2.00 ± 0.10
		5–140	14.0 ± 0.6	0.10± 0.01			33- 140	06.5 ± 0.4	0.06 ± 0.01
	3	0–4	14.0 ± 0.5	3.50 ± 0.13		3	0–10	22.0 ± 0.7	2.20 ± 0.07
		4–140	10.0 ± 0.4	0.07± 0.01			20–140	19.0 ± 0.7	0.16 ± 0.01
	5	0–4	12.0 ± 0.5	3.00± 0.13		5	0–7	25.0± 0.8	3.57 ± 0.11
		6–140	17.0 ± 0.7	0.13± 0.01			30–140	16.0 ± 0.6	0.15 ± 0.01
	7	0–17	27.0± 0.8	1.59± 0.05		7	0–10	27.0 ± 0.9	2.70 ± 0.09
		40–140	17.5 ± 0.7	0.18± 0.01			12–140	14.0 ± 0.6	0.11 ± 0.01

* S1: ASP-2/PE-*co*-VAL/Pδ-VL50; S2: ASP-5/PE-*co*-VAL/Pδ-VL50; S3: ASP-7/PE-*co*-VAL/Pδ-VL50; S4: ASP-10/PE-*co*-VAL/Pδ-VL50.

**Table 7:** Estimated distribution of the cumulative ASP released from ASP/PE-*co*-VAL/Pδ-VL50 drug-carrier systems on the principal digestive organs.

Drug-Carrier System	Stomach Transit (wt%)		Small Intestine Transit (wt%)		Colon Transit (wt%)		Intest/Stomach Ratio (wt/wt)	
Transit time	Min (1 h)	Max (4 h)	Min (4h)	Max (12 h)	Min (48 h)	Max (72 h)	Min (53 h)	Max (88 h)
ASP-2/PE-*co*-VAL/Pδ-VL50	0.60	2.38	1.0	3.36	13.66	15.68	24.43	8.0
ASP-5/PE-*co*-VAL/Pδ-VL50	2.65	13.0	20.8	21.44	16.99	17.38	14.26	2.99
ASP-7/PE-*co*-VAL/Pδ-VL50	3.25	13.0	6.38	7.82	20.65	24.37	8.32	2.48
ASP-10/PE-*co*-VAL/Pδ-VL50	2.10	8.40	10.80	11.68	31.17	34.29	19.98	5.47
